# Enhancement of Mesenchymal Stem Cell-Driven Bone Regeneration by Resveratrol-Mediated SOX2 Regulation

**DOI:** 10.14336/AD.2018.0802

**Published:** 2019-08-01

**Authors:** Yoorim Choi, Dong Suk Yoon, Kyoung-Mi Lee, Seong Mi Choi, Myon-Hee Lee, Kwang Hwan Park, Seung Hwan Han, Jin Woo Lee

**Affiliations:** ^1^Department of Orthopaedic Surgery, Yonsei University College of Medicine, Seoul 03722, South Korea.; ^2^Brain Korea 21 PLUS Project for Medical Science, Yonsei University College of Medicine, Seoul 03722, South Korea.; ^3^Department of Internal Medicine, Brody School of Medicine at East Carolina University, Greenville, North Carolina 27834, USA.; ^4^Severance Biomedical Science Institute, Yonsei University College of Medicine, 50-1 Yonsei -ro, Seodaemun-gu, Seoul 03722, South Korea.; ^5^Lineberger Comprehensive Cancer Center, University of North Carolina-Chapel Hill, Chapel Hill, North Carolina 27599, USA.; ^6^Department of Orthopaedic Surgery, Gangnam Severance Hospital, Yonsei University College of Medicine, Seoul 135-720, South Korea.

**Keywords:** Mesenchymal stem cells, Bone regeneration, Small molecule, Resveratrol, MSC therapy

## Abstract

Mesenchymal stem cells (MSCs) are an attractive cell source for regenerative medicine. However, MSCs age rapidly during long-term *ex vivo* culture and lose their therapeutic potential before they reach effective cell doses (ECD) for cell therapy. Thus, a prerequisite for effective MSC therapy is the development of cell culture methods to preserve the therapeutic potential during long-term *ex vivo* cultivation. Resveratrol (RSV) has been highlighted as a therapeutic candidate for bone disease. Although RSV treatment has beneficial effects on bone-forming cells, *in vivo* studies are lacking. The current study showed that long-term (6 weeks from primary culture date)-cultured MSCs with RSV induction retained their proliferative and differentiation potential despite reaching ECD. The mechanism of RSV action depends entirely on the SIRT1-SOX2 axis in MSC culture. In a rat calvarial defect model, RSV induction significantly improved bone regeneration after MSC transplantation. This study demonstrated an example of efficient MSC therapy for treating bone defects by providing a new strategy using the plant polyphenol RSV.

Mesenchymal stem cells (MSCs) have the capacity for self-renewal and differentiation into bone, adipose tissue, and cartilage [1]. Thus, they have been widely used to treat skeletal diseases including osteogenesis imperfecta [2, 3] and osteoarthritis [4, 5]. For clinical application, it is necessary to expand these cells into therapeutic doses for several passages *in vitro* because the cell numbers obtained directly from patients are too small for clinical applications. In addition, MSCs undergo cellular senescence with telomere shortening and lose their stem cell characteristics during expansion* in vitro* [6-8]. Therefore, a major challenge to the therapeutic use of MSCs is maintaining their self-renewal capacity and multipotency during long-term *in vitro* cultivation to reach effective cell doses required for clinical application.

Resveratrol (RSV) is a naturally occurring polyphenolic compound with beneficial biological effects such as antiaging [9], antioxidant [10], and bone protective activities [11], indicating its potential as a therapeutic candidate. We previously optimized methods for culturing MSCs *in vitro* with RSV such as the concentration and duration of RSV treatment and procedure for passaging the cells [12]. In the study, we further evaluated the safety and efficacy of using MSCs cultured with RSV for MSC therapy. Although many studies have reported the effects of RSV in MSCs [13-15], no study has confirmed the efficacy of RSV in bone healing *in vivo*. Therefore, the aim of this study was to examine RSV application for MSC-based therapy in the preclinical setting, particularly in bone regeneration. In this study, we confirmed that long-term treatment with 1-μM RSV retained the proliferative and differentiation potential of MSCs during prolonged *ex vivo* culture until effective cell doses (ECD) were reached for cell therapy. We designated the MSCs reaching ECD (1 x 10^7^ cells) as 6w-MSCs, since it took about 6 weeks to acquire the ECD cell number. Transplantation of 6w-MSCs with RSV induction clearly enhanced the bone regeneration potential in a rat model with calvarial defects. Therefore, MSCs expanded via cell culture with RSV induction are an efficient cell source for stem cell therapy in regenerative medicine.

## MATERIALS AND METHODS

### Isolation, cultivation, and RSV treatment

Bone marrow aspirates were obtained from the posterior iliac crests of six adult donors with the approval of institutional review board (IRB) of Yonsei University College of Medicine (IRB No. 4-2017-0232), and written informed consent was obtained from all patients. All methods were performed according to relevant guidelines and regulations of the institution. MSCs isolated from bone marrow were selected and cultured in accordance with published protocols [16], and their characteristics *(i.e.*, positivity for CD90 and CD105, and negativity for CD34 and CD45) were confirmed by flow cytometry. MSC characteristics of the harvested cells were confirmed in our previous reports [16]. All MSCs used in this study were isolated and cultured individually. Each experimental result was confirmed in at least three donor samples as well as triplicates. MSCs were maintained in low-glucose Dulbecco’s modified Eagle’s medium (DMEM-LG; Invitrogen, Grand Island, NY, USA) that was supplemented with 10% fetal bovine serum (FBS; Gibco, Grand Island, NY, USA) and 1% antibiotic-antimycotic solution (Invitrogen) at 37 °C in 5% CO_2_ atmosphere. The cells were grown to 80% confluence, and then detached by incubation with 0.25% trypsin /EDTA (Invitrogen) centrifuged at 188 g for 3 min. The cells collected by centrifugation were re-plated at density of 2 x 10^5^ cells, and subcultivation was continued until the number of cells reached 1 x 10^7^ cells. The concentration of RSV (Sigma-Aldrich, St. Louis, MO, USA) used (1 µM) was selected according to a previous study [12]. MSC cultivation was continued without freezing or terminating the culture until the number of cells reached 1x10^7^ cells. Cells were subcultured every 3 days, and then counted each time. At each subculturing session, 2 x 10^5^ MSCs were seeded onto new 10-cm^2^ culture dishes. The number of MSCs was recorded every time the cells were subcultured, and the numbers were accumulated until we had approximately 1x10^7^ cells; after that, the cell culture was terminated. The period lasted for about 6 weeks. The passage number, which reached 1 x 10^7^ cells, can be somewhat different for each MSC derived from different donors. Therefore, the number of each passage requiring 1 x 10^7^ cells has been recorded in each figure legend.

### Western blotting

MSCs were lysed in passive lysis buffer (Promega, Madison, WI, USA). Protein concentrations were determined using Bio-Rad Protein Assay (Bio-Rad Laboratories, Inc., Hercules, CA, USA), and 30 mg of protein per lane was analyzed by 10% sodium dodecyl sulfate-polyacrylamide gel electrophoresis (Sigma-Aldrich). After the proteins were transferred to polyvinylidene fluoride membranes, membranes were blocked with 5% skim milk (BD Biosciences, Sparks, MD, USA) or 5% BSA (Sigma-Aldrich) for 1 h at room temperature. Membranes were incubated for about 12 h with antibodies against SIRT1 (Santa Cruz Biotechnology, Dallas, TX, USA), SOX2 (Abcam, Cambridge, UK), OCT4 (Santa Cruz Biotechnology), NANOG (BD Biosciences), CASPASE-3 (Cell Signaling Technology, Danvers, MA, USA), Cleaved CASPASE-3 (Cell Signaling Technology), P53 (Santa Cruz Biotechnology), P21 (Santa Cruz Biotechnology), P16 (Abcam), and HSP90 (Santa Cruz Biotechnology), which served as a loading control.

### Colony-forming unit fibroblast assay

MSCs were seeded at 1 × 10^3^ cells in 100-mm culture dishes and maintained in DMEM-LG supplemented with 20% fetal bovine serum for 12 days. The cells were fixed in 1:1 acetone:methanol, stained with 20% crystal violet solution (Merck, Darmstadt, Germany) for 10 min in the dark, and washed with distilled water. The colony-forming ability of the stained cells was evaluated for three donors in triplicate.

### Cell proliferation assay

Proliferative capacity of 6w-MSCs was examined using EZ-Cytox Kit (Daeil Lab Service, Seoul, Korea). The 6w-MSCs were seeded in 12-well culture plate at density of 1 × 10^4^ cells per well. DMEM-low glucose containing 10% FBS medium was used for maintaining cells for 7 days, and culture media were replaced every 2 days during assay periods. To measure levels of cell proliferation, cells were washed with PBS, and 20 μl of EZ-Cytox (tetrazolium salts) solution was added to each well and incubated at 37 °C for 4 h. After incubation, the conditioned media were transferred to 96-well plate. Absorbance was measured at 450 nm. All samples were tested in triplicate (n =3).

### Osteogenic and adipogenic differentiation

The materials and methods for MSC differentiation, staining, and quantitative analysis were described previously [16]. Briefly, MSCs were seeded at 8 X 10^4^ cells per well in 12-well culture plates. For osteogenic differentiation, cells were maintained for 10 days in osteogenic medium [DMEM-low glucose medium containing 10% FBS, 1% antibiotic-antimycotic solution, 100 nM dexamethasone (Sigma), 10 mM β-glycerophosphate (Sigma), and 50 μg per ml ascorbic acid (Gibco)]. Alizarin red S staining was employed to determine osteogenic differentiation activity. For alizarin red S staining, after being fixed in ice-cold 70% ethanol, 1 ml of freshly prepared 3% alizarin red S solution (wt/vol) (Sigma) was added, then incubated in the dark for 30 minutes. For quantitative analysis of alizarin red S, absorbance was detected at 595 nm after destaining with 10% cetylpyridinium chloride monohydrate (Sigma) for 30 minutes. For adipogenic differentiation, cells were maintained for 10 days in adipogenic medium [DMEM-low glucose medium containing 10% FBS, 1% antibiotic-antimycotic solution, 1 μM dexamethasone, 0.5 mM isobutyltethylxanthin (Sigma), 5 μg / ml insulin (Gibco), and 200 lM indomethasin (Sigma)]. Oil red O staining was employed to determine adipogenic differentiation activity. To stain lipid droplets by oil red O, after being fixed in 10% neutral buffered formalin, 1 ml of 0.18% oil red O solution (Sigma) was added and incubated for 30 minutes. For quantitative analysis of oil red O-stained cells, absorbance was detected at 500 nm after destaining with 100% isopropanol for 30 minutes.

### RNA interference

Negative control and SIRT1 siRNAs (siRNA No. 1137490) were purchased from Bioneer (Daejeon, South Korea, www.sirna.bioneer.co.kr). Negative control-sense siRNA targeted sequence 5′-CCUACGCCACCAAUUU CGU-3′, and negative control-antisense siRNA targeted sequence 5′-ACGAAAUUGGUGGCGUAGG-3′. SIRT1 -sense siRNA targeted sequence 5′-CUGUGAAAU UACUGCAAGA(dTdT)-3′, and SIRT1-antisense siRNA targeted sequence 5′-UCUUGCAGUAAUUUCACA G(dTdT)-3′. Briefly, MSCs treated with RSV (1 μM) were plated to obtain 70% confluence in 6-well plates and transfected with 100 nM of negative control or SIRT1 siRNA using Lipofectamine LTX (Invitrogen). After 6 h of transfection, growth medium was added.

### Immunoprecipitation

Cell lysates from MSCs were prepared with non-denaturing lysis buffer, as previously described [16]. Lysates were incubated with protein A/G agarose beads (Santa Cruz Biotechnology) and antibodies against acetylated lysine (Cell Signaling Technology) and SOX2 (Abcam). Beads conjugated with both lysates and antibodies were collected after centrifugation and washed three times with lysis buffer. The complexes were released from beads by boiling with 2× SDS sample dye, and then western blotting was performed. All membranes were incubated with antibody against SOX2 (Abcam) for 12 h, followed by incubation with HRP-conjugated secondary antibody for 1 h.

### Immunocytochemistry

Immunostaining was performed according to our previously reported protocol [16]. MSCs were seeded at 2 000 cells/cm^2^ on 4-well glass chamber slides (Nalge Nunc International, Rochester, NY, USA), and cells were incubated in 5% CO_2_ incubator at 37 °C overnight. After that, cells were washed with PBS, followed by fixation with 4% paraformaldehyde (Sigma-Aldrich) for 30 min. Permeabilization was accomplished with 1% Triton X-100 in PBS for 10 min, followed by blocking with 3% bovine serum albumin (BSA) in PBS for 1 h. Cells were incubated with a 1:100 dilution of primary antibodies against SOX2 (Abcam), SIRT1 (Santa Cruz Biotechnology), and bromodeoxyuridine (Santa Cruz Biotechnology) overnight at 4 °C. After washing three times with PBS, cells were incubated with fluorescein isothiocyanate (FITC)-, phycoerythrin (PE, red)-, or alexa fluor 568 (Yellow)-conjugated secondary antibodies (Santa Cruz Biotechnology) at a 1:5000 dilution in PBS containing 1% BSA for 1 h at room temperature in the dark. The nuclei were stained with 4′,6-diamidino-2-phenylindole (Sigma-Aldrich), and then examined by LSM780 scanning laser confocal microscope (Zen 2012; Carl Zeiss MicroImaging GMBH, Jena, Germany).

**Figure 1.**
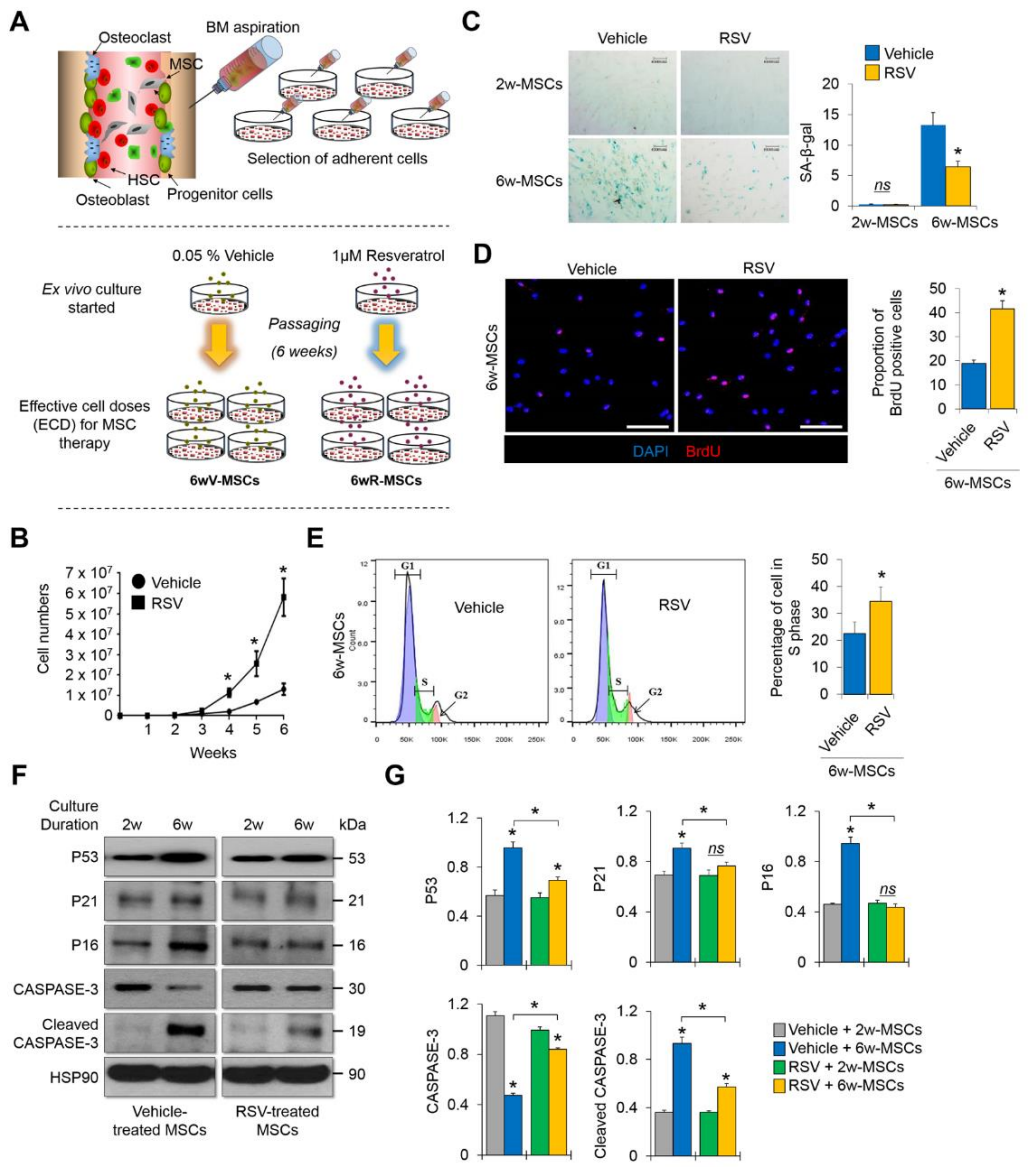
6w-MSCs with RSV induction preserve the proliferative capacity even after reaching effective cell dose (ECD) for MSC therapy (**A**) Scheme for MSC isolation and long-term cultivation to obtain ECD-MSCs. First, 0.05% vehicle (EtOH) or 1 μM RSV was added to the medium for MSC culture and the vehicle or RSV-containing medium was exchanged every 2 days. The cells were cultured until vehicle-treated MSCs reached 1 × 10^7^ cells, generally regarded as the ECD for bone regeneration. (**B**) The numbers of MSCs between 1-6 weeks of expansion in the absence or presence of RSV (*n* = 6 donors). The cells were counted every 7 days. (**C**) SA-β-gal assay was performed to compare cellular senescence between vehicle and RSV groups. 2w-MSCs refers to the MSCs cultured during 2 weeks from primary culture date, and 6w-MSCs refers to the MSCs cultured during 6 weeks from primary culture date in order to obtain 1 × 10^7^ cells. SA-β-gal-positive cells were quantitated by ImageJ (*n* = 3, in triplicate per donor) (right). *p < 0.05 compared to vehicle-treated MSCs. 2w-MSCs, 3rd passage; 6w-MSCs, 13th passage. (**D**) Immunocytochemistry was performed to observe the bromodeoxyuridine-positive cell portion. Nuclei were stained with 4′,6-diamidino-2-phenylindole and images were captured by confocal microscopy. Scale bar = 100 µm. *, *p* < 0.05 compared to vehicle-treated 6w-MSCs (*n* = 3, in triplicate per donor). 6w-MSCs, 13th passage. (**E**) The proportion of 6w-MSCs treated with vehicle or RSV in each cell cycle phase was evaluated by flow cytometry with propidium iodide staining. *, *p* < 0.05 compared to vehicle-treated 6w-MSCs (*n* = 3, in triplicate per donor). 6w-MSCs, 13th passage. (**F**) Protein levels of P53, P21, P16, CASPASE-3, and cleaved CASPASE-3 were quantified by western blot analysis and normalized to that of HSP90. 2w-MSCs, 4th passage; 6w-MSCs, 13th passage. (**G**) Quantification of each protein level was determined by GraphPad Prism software (version 6.0). *, *p* < 0.05 compared to 2w- or vehicle-treated MSCs (*n* = 3, in triplicate per donor).

### SOX2 overexpression

Information for the *SOX2* has been described previously[16]. Briefly, *SOX2* cDNA was inserted into the pEGFP-C1 vector between SacI and KpnI (Takara Bio, Inc., Shiga, Japan) to generate pEGFPC1/SOX2, which expresses a green fluorescent protein (GFP)-SOX2 fusion protein. For SOX2 overexpression, MSCs were plated to obtain 70% confluence in six-well plates and transfected with pEGFP-C1/SOX2 using Lipofectamine LTX (Invitrogen).

### Cell cycle analysis

Single-cell suspension of differentiated cells was prepared as previously described [17]. MSCs treated with ethanol (vehicle) or RSV were harvested by incubation with 0.25% trypsin/EDTA and washed twice in PBS. Cells from each group (1 × 10^6^) were fixed in ice-cold 70% ethanol for 30 min, stained with 50 µg/ml propidium iodide (Sigma-Aldrich) containing 100 µg/ml RNase A (Sigma-Aldrich) for 15 min at 4 °C, and then analyzed using FACSCalibur™ instrument (BD Biosciences, San Jose, CA, USA) to detect the cell cycle distribution. All samples used were tested in triplicate (*n* = 3).

### Quantitative real-time polymerase chain reaction (qRT-PCR)

Total RNA was isolated using an RNeasy kit (Qiagen, Hilden, Germany). Total RNA (1 mg) was then reverse-transcribed using an Omniscript^®^ kit (Qiagen). Quantitative real-time polymerase chain reaction was performed as previously described [16]. Primer sets were validated and purchased from Bioneer (https://us.bioneer.com/products/rnai/rnaioverview.aspx, Daejeon, South Korea). The primer sets and numbers used were as follows: *Glyceraldehyde 3-phosphate dehydrogenase* (*GAPDH*, P267613), *runt-related transcription factor 2* (*RUNX2*, P229954), *collagen, type I, alpha 1* (*COL1A1*, P157768), *peroxisome proliferator-activated receptor gamma* (*PPAR-γ*, P102309), *adiponectin* (*APN*, P160254), *cyclin A* (*CCNA*, P212796), *cyclin D* (*CCND*, P298560), *cyclin-dependent kinase 2* (*CDK2*, P136765), *and cyclin E* (*CCNE*, P220201).

### Senescence-associated-β-galactosidase assay (SA-β-gal assay)

The SA-β-gal assay was performed using a cellular senescence assay kit (Millipore, Billerica, MA, USA) following the manufacturer’s instructions. Cells were washed with PBS, and then were fixed for 10 min at room temperature with 1X fixing solution. After washing with D.W, cells were stained with prepared 1X SA-β-gal detection solution over 4h in the dark at 37 °C incubator without CO2. The number of positive cells was quantified by Image J software, version 1.41 (National Institutes of Health, Bethesda, MA, USA).

### Rat calvarial defects

All animal use procedures were conducted with the approval of the Institutional Animal Care and Use Committee of Yonsei University College of Medicine (Approval number: IACUC-2016-0099). Twelve-week-old male Sprague-Dawley rats were anesthetized by an intraperitoneal injection of Zoretile (30 mg/kg body weight) and xylazine (10 mg/kg body weight). After shaving the hair on the head, a longitudinal incision was made in the skull, and then critical-sized calvarial bone defects with a diameter of 8 mm were created using a trephine bur. The defects were irrigated with saline and MSCs (1 × 10^6^/defect) mixed with fibrin glue were implanted into the defects, followed by suturing of the soft tissue. For pain relief, rats were administered a subcutaneous injection of meloxicam (0.2 mg/kg body weight). At 4-8 weeks after operation, all rats tested were sacrificed and the skull was harvested for micro-computed tomography (µCT) and histological analysis (*n* = 10 per a group).

### µCT analysis

After fixation in 10% formalin for 5 days, the skulls were scanned with a high-resolution µCT instrument (SkyScan 1076; Bruker, Billerica, MA, USA) to quantitatively evaluate calvarial bone regeneration at the defect site as described previously[18]. Briefly, the imagery was reconstructed and analyzed using NRecon v1.6.6.0 and CTAn v1.13.2.1 (Bruker), respectively. Three-dimensional model visualization software CTVol v2.0 (Bruker) was used to analyze calvarial bone regeneration. The settings for X-ray source were 70 kVp voltage and 140 mA current, and 0.5-mm-thick aluminum filter was used for beam induration. Pixel size was 18 mm, exposure time was 1475 ms, and rotation step was 0.5°, with a complete rotation through 360°.

### Histological analysis and immunohistochemistry

The skulls were fixed for 1 week in 10% formalin and then embedded in paraffin. The paraffin-embedded sections were deparaffinized, rehydrated, and washed three times with PBS, and then the sections were used to evaluate tissue repair in the damaged regions. Histological analysis and immunohistochemistry were performed as previously described[18]. Briefly, tissue samples were sliced at a thickness of 4 µm and stained with hematoxylin and eosin (H&E) to observe new bone formation or incubated with human anti-vimentin antibodies (Santa Cruz Biotechnology, 1:100 dilution) to confirm whether the regenerated bone tissues were derived from human origin. Human vimentin was detected with a secondary goat anti-mouse IgG-horseradish peroxidase antibody (GenDEPOT, Katy, TX, USA) and 3,3′-diaminobenzidine (Vector Laboratories, Burlingame, CA, USA). The stained samples were observed using VS120 virtual microscope (Olympus, Tokyo, Japan), and sample images were analyzed using OlyVIA 2.5 software (Olympus). For immunofluorescence, paraffin-embedded tissue sections with a thickness of 4 µm were deparaffinized,

**Figure 2.**
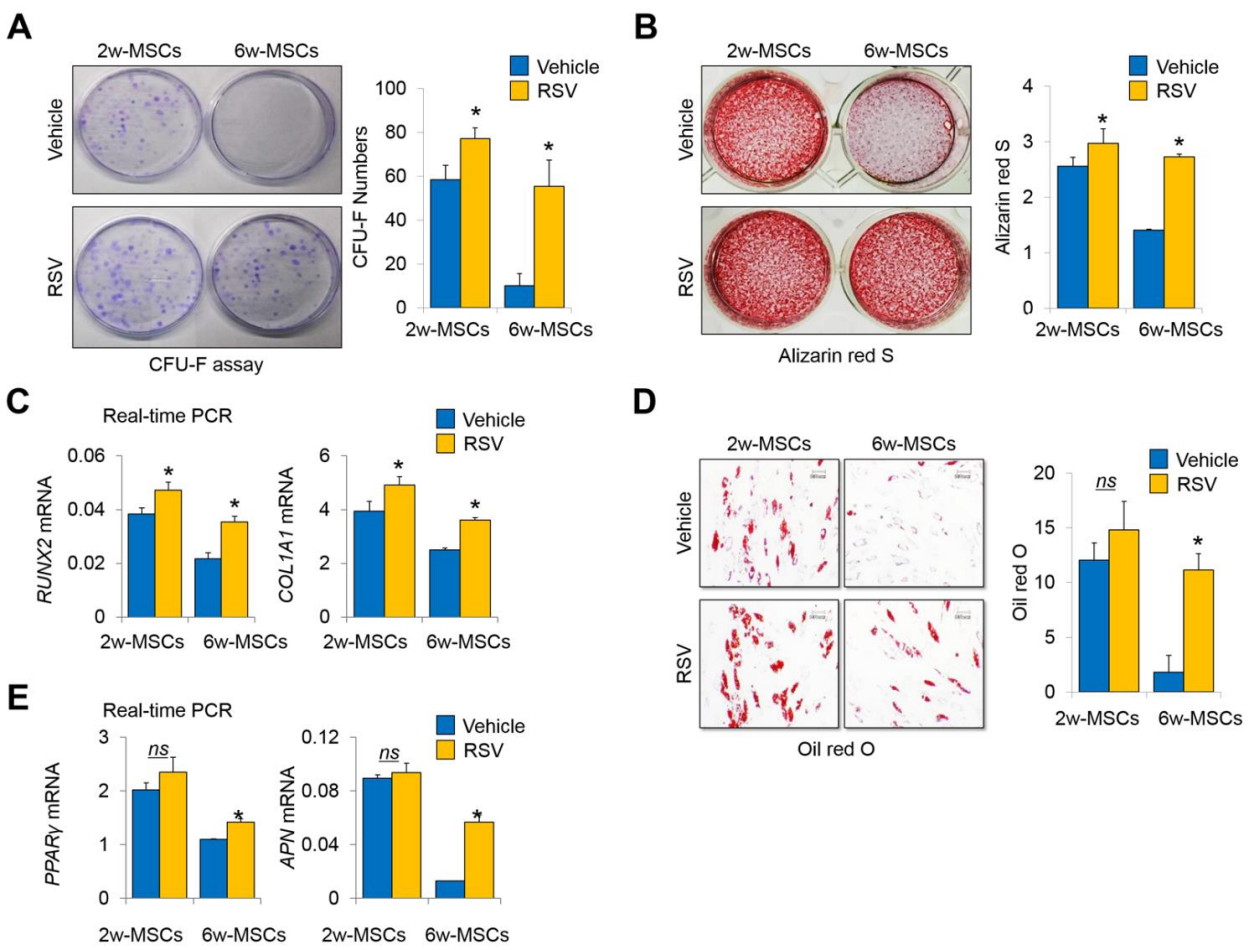
Long term-cultured MSCs with RSV induction preserve the self-renewal and multi-differentiation capacities even after reaching ECD for MSC therapy (**A**) 2w- and 6w-MSCs (1 × 10^3^ cells per well in 100-mm dishes) treated with vehicle or RSV were incubated in growth medium for 12 days. The colony-forming abilities of the cells were compared by crystal violet (CV) staining. The colony number was counted by three independent observers (*n* = 3, in triplicate per donor). *, *p* < 0.05. 2w-MSCs, 2nd passage; 6w-MSCs, 14th passage. (**B**) Alizarin red S staining was performed to detect mineral deposition and was quantified with ImageJ software (*n* = 3, in triplicate per donor). *, *p* < 0.05 compared to vehicle. 2w-MSCs, 2nd passage; 6w-MSCs, 14th passage. (**C**) The mRNA expression of *RUNX2* and *COL1A1* was determined in 2w- or 6w-MSCs treated with 0.05% vehicle (EtOH) or 1-μM RSV by real-time quantitative PCR (*n* = 3, in triplicate per donor). *, *p* < 0.05 compared to vehicle. 2w-MSCs, 3rd passage; 6w-MSCs, 14th passage. (**D**) Oil red O staining was performed to detect lipid droplets and were quantified with ImageJ software (*n* = 3, in triplicate per donor). *, *p* < 0.05 compared to vehicle. 2w-MSCs, 2nd passage; 6w-MSCs, 14th passage. (**E**) The mRNA expression of *PPARγ* and *ADIPONECTIN* was determined in 2w- or 6w-MSCs treated with 0.05% vehicle (EtOH) or 1-μM RSV by real-time quantitative PCR (*n* = 3, in triplicate per donor). *, *p* < 0.05 compared to vehicle. 2w-MSCs, 3rd passage; 6w-MSCs, 14th passage.

rehydrated, and washed twice with PBS. To reduce nonspecific background staining due to endogenous peroxidase, sections were incubated in hydrogen peroxide blocks for 10 min and washed twice with PBS. Sections were incubated with SIRT1 (Abcam), SOX2 (Abcam), RUNX2 (Millipore), or OSTEOCALCIN (Abcam) antibodies with human VIMENTIN antibody (Santa Cruz Biotechnology) overnight at 4 °C, and then washed with PBS. Phycoerythrin-conjugated goat anti-rabbit secondary antibodies (Santa Cruz Biotechnology) and FITC-conjugated goat anti-mouse secondary antibodies were used to visualize the primary antibodies. All primary and secondary antibodies were applied at dilutions of 1:100 and 1:5000, respectively. The nuclei were stained with 4′,6-diamidino-2-phenylindole (DAPI, Sigma). Images were examined using Zeiss LSM 700 confocal laser scanning microscope (ZEN 2012 software; Carl Zeiss Micro Imaging GmbH, Jena, Germany).

### Chromosomal abnormality assay

To analyze chromosome instability, 20 cells at metaphase per sample were counted after staining. For karyotyping, at least 10 more cells at metaphase were analyzed and images were captured. GTG-(Giemsa-Trypsin)-banding with 525 bands of resolution was performed by Samkwang medical laboratories (www.smlab.co.kr/).

### In vivo tumorigenicity assay

To evaluate *in vivo* tumorigenicity, MSC, RSV-MSC and NKM-74 cells (5 × 10^6^) were suspended in 200 µL of PBS containing 20% Matrigel (Sigma), and subcutaneously injected into Balb/C/nu/nu mice (Orient Bio, Inc., Seongnam, Korea). One or five months after implantation, tumor dimensions were determined with a caliper, and the volumes were calculated using the following formula: Tumor volume = π/6 × length × width × height [19]. Length represents the largest tumor diameter, and width represents the perpendicular tumor diameter.

### Statistical analyses

To determine the differences between two groups, Student’s *t*-tests were performed. For more than two groups, one-way analysis of variance was performed if normality tests passed, followed by Tukey’s multiple comparison tests for all pairs of groups. The data are presented as the mean ± standard deviation. All experiments were conducted in triplicate. GraphPad Prism software (version 6.0; GraphPad, La Jolla, CA, USA) was used for statistical analysis. Values of *p* < 0.05 were considered statistically significant.

## RESULTS

### RSV allows mass production of MSCs with effective cell doses (ECD)

The marrow mixture was isolated and seeded onto a culture plate to allow the MSCs to attach the plastic culture dish. Attached cells were divided into groups treated with 0.05% vehicle or 1 μM RSV and cultured until vehicle-treated MSCs reached an ECD (1 x 10^7^ cells) (Fig. 1A). There have been many clinical trials using MSCs to treat small or critical bone defects[20]. A minimum number of cells is required to obtain a successful therapeutic effect following MSC implantation. To date, there is no standard ECD for treating bone defects, but a minimum of 1 x 10^7^ cells appears to be required for treating bone defects (See supplementary Table 1). Thus, we grew control MSCs treated with vehicle to 1 x 10^7^ cells, and then compared the obtained cell amounts with RSV-treated MSCs. In the absence of RSV, the cell number reached more than 1 × 10^7^ at about 6 weeks, whereas in the presence of RSV, the cell number was approximately 1 x 10^7^ at about 4 weeks. Indeed, at 6 weeks, the number of MSCs cultured in the presence of RSV (5.82 ± 0.92 × 10^7^) was approximately 4-fold higher than that of MSCs cultured in the absence of RSV (1.31 ± 0.29 × 10^7^) (Fig. 1B, supplementary Table 2). We designated these cells as 6w-MSCs (MSCs with an effective cell dose for bone regeneration) and used the cells for further experiments. It took about 2 weeks to obtain a sufficient number of young MSCs for further experiments. Although these young MSCs were not suitable for *in vivo* experiments due to the limited number of cells, they were sufficient for further *in vitro* experiments. Therefore, we also designated them as 2w-MSCs (not effective dose for *in vivo* bone regeneration, but appropriate cell number for *in vitro* test), and used the cells as control groups of 6w-MSCs. We next compared the degree of cellular senescence between vehicle- and RSV-treated 6w-MSCs by SA-β-galactosidase (SA-β-gal) staining. RSV alleviated the level of cellular senescence compared to that of its counterpart (Fig. 1C). In addition, the proportion of bromodeoxyuridine-positive cells was higher in 6w-MSCs with RSV induction than in its counterpart (Fig. 1D). Moreover, the cell populations of control 6w-MSCs in S phase were smaller than those of 6w-MSCs with RSV induction (Fig. 1E). The expression of cell cycle-related genes *CCNA*, *CCND*, *CDK2*, and* CCNE*, which are associated with S phase [21, 22], was higher in 6w-MSCs with RSV induction compared to in control 6w-MSCs (Supplementary Fig. 1). However, the results of SA-β-gal assay and cell cycle analysis cannot fully explain the higher rate of growth by RSV treatment in MSC culture. To address this phenomenon more clearly, we checked the protein level of CASPASE-3,

**Figure 3.**
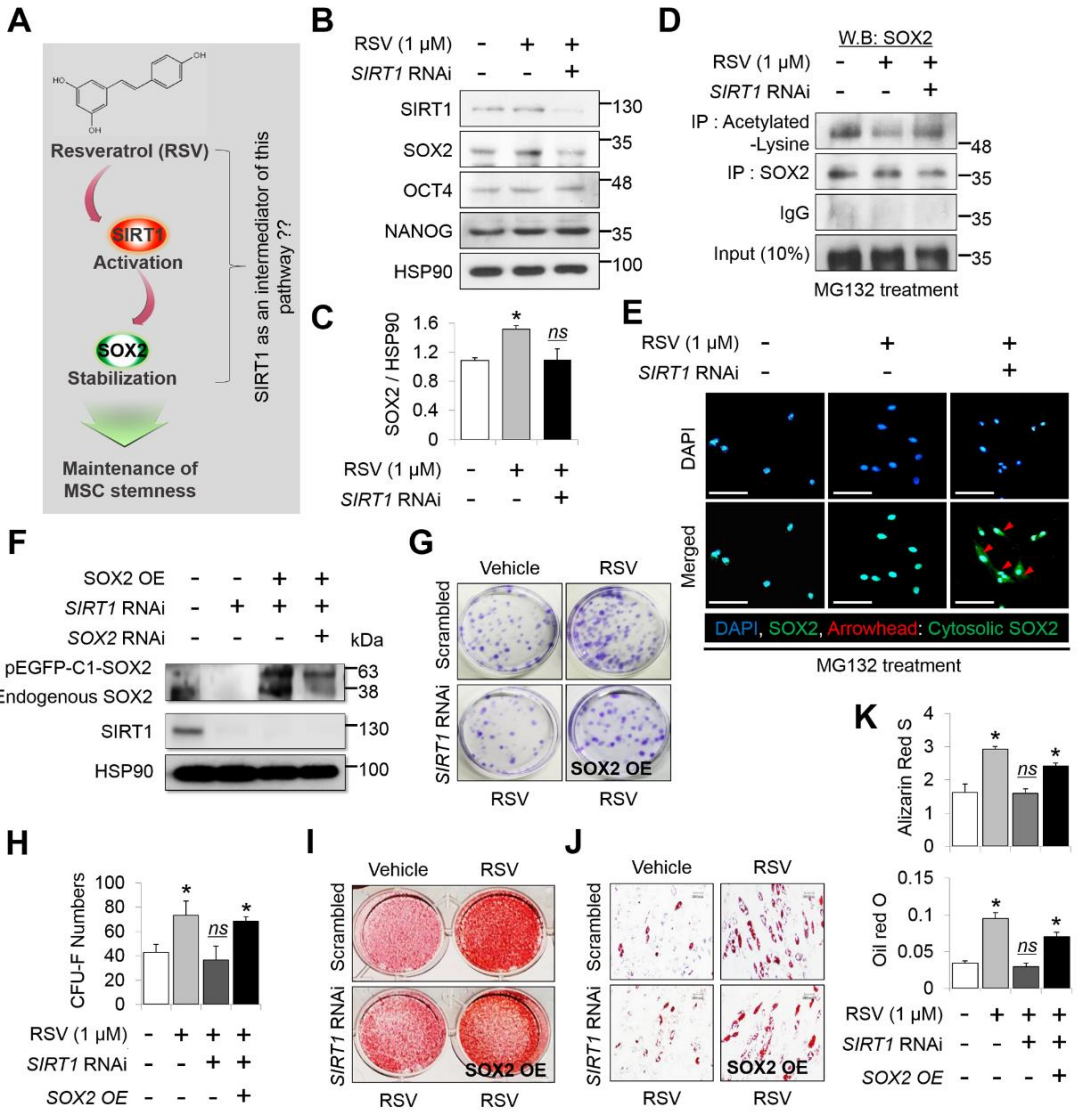
RSV treatment stabilizes SOX2 protein levels in MSCs depending on the presence of SIRT1 (**A**) Expected model of the mechanism of RSV action on the regulation and maintenance of MSC stemness via SIRT1-SOX2 axis. (**B**) Protein levels of SIRT1, SOX2, OCT4, and NANOG were quantified by western blot analysis and normalized to that of HSP90. (**C**) Quantitative analysis of SOX2 protein was performed by ImageJ software (*n* = 3, in triplicate per donor). *, *p* < 0.05, *ns*; not significant compared to control. MSCs of 4th passage were used for this western blot. (**D**) Immunoprecipitation was conducted in the presence of MG132. To confirm acetylated-lysine and SOX2, each protein was immunoprecipitated using antibodies targeting each protein followed by western blotting using an anti-SOX2 antibody (*n* = 3, in triplicate per donor). All study groups were treated with MG132 (10 µM), a proteasome inhibitor. MSCs of 4th passage were used for immunoprecipitation. (**E**) Immunocytochemistry was conducted to observe cellular localization of SOX2 from the nucleus to the cytoplasm, following SIRT1 knockdown in the presence of RSV (*n* = 3, in triplicate per donor). To inhibit proteasomal degradation of SOX2, all study groups were treated with MG132 (10 µM). Nucleus was stained with DAPI, and images were captured by confocal microscopy. Red arrowhead indicates nuclear exports of SOX2 protein. Scale bar = 50 μm. MSCs of 4th passage were used for immunocytochemistry. (**F**) Efficiency of SIRT1 knockdown or SOX2 overexpression was confirmed by western blot analysis in MSCs (*n* = 3, in triplicate per donor). MSCs of 4th passage were used for this western blot. (**G**) Colony-forming cells were detected by CV staining, and (H) the number was counted by three observers (lower panel) (*n* = 3, in triplicate per donor). *, *p* < 0.05. Alizarin red S (I) and oil red O (J) staining was conducted to compare mineralization and accumulation of lipid droplets. (**K**) Quantitative analysis for mineralization was measured at 595 nm absorbance (lower panel). *, *p* < 0.05. Lipid droplets were quantified with ImageJ software (*n* = 3, in triplicate per donor). Scale bar = 60 µm. *, *p* < 0.05. MSCs of 12th passage were used for CV, alizarin red S, and oil red O stains.

which is known as a family of endoproteases that provide critical links in cell regulatory networks controlling the cell cycle, cell proliferation, and anti-apoptotic capacity of MSCs[23]. We also checked the protein levels of markers that are related to the cell cycle and potential cellular senescence, such as P53, P21, and P16[24]. In our results, RSV treatment prevented 6w-MSCs from cleavage of CASPASE-3 protein as well as accumulation of cell cycle inhibitors (Fig. 1F, 1G), indicating that there were more proliferating and healthy cells in 6w-MSCs with RSV induction than in control 6w-MSCs.

### 6w-MSC with RSV induction retain self-renewal and multipotency over multiple passages

The colony-forming unit fibroblast assay showed that 6w-MSCs with RSV induction retained their colony-forming abilities compared to control 6w-MSCs (Fig. 2A). To compare multipotency, we induced osteogenic and adipogenic differentiation of MSCs. As expected, 6w-MSCs with RSV induction maintained their potential for osteogenic and adipogenic differentiation. Alizarin red S staining showed that osteogenic differentiation was significantly more pronounced in 6w-MSCs with RSV induction compared to in control 6w-MSCs (Fig. 2B). The mRNA levels of *RUNX2* and *Collagen Type I Alpha 1* (*COL1A1*) were also highly expressed in 6w-MSCs treated with RSV (Fig. 2C). The 6w-MSCs with RSV induction also maintained their ability to differentiate into the adipogenic lineage. Oil red O staining showed that 6w-MSCs treated with RSV formed lipid droplets more frequently than control 6w-MSCs upon adipogenic induction (Fig. 2D). The mRNA levels of *PPARγ* and *Adiponectin* (*APN*) were also highly expressed in 6w-MSCs treated with RSV (Fig. 2E). To test a possibility that SOX2 overexpression solely regulated the self-renewal and multipotency of 6w-MSCs independently of RSV-mediated signaling, we compared the effect of single SOX2 overexpression with its counterpart (pEGFP-C1 vector only) with or without RSV. SOX2 overexpression enhanced the proliferative capacity of vector control MSCs, and RSV synergistically increased the proliferative capacity of MSCs overexpressing SOX2 (supplementary Fig. 2A and 2B). Likewise, RSV enhanced the osteogenic and adipogenic potentials of 6w-MSCs overexpressing SOX2 (supplementary Fig. 2C-2F), which implies that RSV and SOX2 may have a synergistic effect in MSC self-renewal and multipotency. These data suggest that 6w-MSCs with RSV induction retain their self-renewal capacity and multipotency during *ex vivo* expansion over multiple passages.

### SIRT1 and SOX2 are required for 6w-MSCs with RSV induction to maintain stemness

We previously reported that SIRT1 positively regulated SOX2 to maintain the stemness of early passage-MSCs and RSV-induced enhancement of MSC proliferation and differentiation was largely dependent on the presence of SOX2[16]. However, we did not determine whether RSV-mediated SOX2 overexpression depends on SIRT1 in the study (Fig. 3A). To clarify whether RSV-mediated SOX2 activation requires SIRT1, *SIRT1* was knocked down in RSV-treated MSCs and then western blot analysis was performed. The results showed that the SOX2 protein level increased by RSV treatment was significantly reduced in the *SIRT1* RNAi group, while the levels of OCT4 and NANOG, known to be important regulators for maintaining MSC stemness, were not altered by RSV treatment or *SIRT1* RNAi (Fig. 3B, 3C). SIRT1 physically interacts with SOX2 protein to deacetylate and prevent nuclear export of the protein[16]. This mechanism is well-conserved in mouse somatic reprogramming[25]. We confirmed that RSV induced deacetylation of SOX2 and *SIRT1* RNAi abolished the RSV-mediated effect on SOX2 protein (Fig. 3D). Additionally, *SIRT1* RNAi induced nuclear export of SOX2 under RSV stimulation (Fig. 3E). To confirm whether RSV-mediated SOX2 activation requires SIRT1 as an intermediator and is important for enhancing MSC stemness, we used the pEGFP-C1 SOX2 vector to express SOX2-GFP in the presence of RSV and *SIRT1* RNAi (Fig. 3F). As a result, RSV enhanced MSC self-renewal and multi-potency, while *SIRT1* RNAi completely abolished the RSV effects (Fig. 3G-3K). As expected, SOX2 overexpression rescued the effects of *SIRT1* RNAi in the presence of RSV (Fig. 3G-3K). These results indicate that SIRT1 acts as an intermediator of RSV-mediated SOX2 activation, and the SIRT1-SOX2 axis is critical for the RSV-mediated benefit in maintaining MSC stemness.

### RSV induction preserves nuclear levels of SIRT1 and SOX2 proteins during 6w-MSC proliferation and differentiation

**Figure 4.**
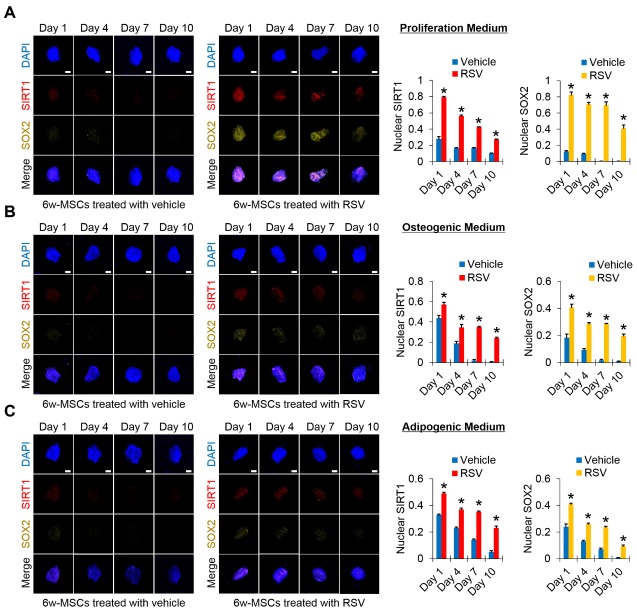
Time-course patterns of RSV-mediated SIRT1-SOX2 regulation in 6w-MSCs (**A**) Immunofluorescence was performed to observe the time course patterns of RSV-mediated SIRT1-SOX2 regulation in the vehicle- and RSV-treated 6w-MSCs cultured under basal DMEM-low glucose medium containing 10% FBS and 1% antibiotic and antimycotic. SIRT1 and SOX2-stained cells were analyzed using Image J software. (**B**) Immunofluorescence was performed to observe the time course patterns of RSV-mediated SIRT1-SOX2 regulation in the vehicle- and RSV-treated 6w-MSCs cultured under osteogenic medium. SIRT1 and SOX2-stained cells were analyzed using Image J software. (**C**) Immunofluorescence was performed to observe the time course patterns of RSV-mediated SIRT1-SOX2 regulation in the vehicle- and RSV-treated 6w-MSCs cultured under adipogenic medium. SIRT1 and SOX2-stained cells were analyzed using Image J software. The nucleus was stained with DAPI, SIRT1was stained with phycoerythrin (PE, red)-conjugated secondary antibody, and SOX2 was stained with alexa fluor 568 (Yellow)-conjugated secondary antibody. Scale bar=10 μm. 6w-MSCs of 11th passage were used for this immunofluorescence.

SOX2 plays a critical role in the improvement of cell proliferation and multipotency, and preserving SOX2 expression in MSCs can be a useful method for maintaining MSC population with stemness for long-term culture periods [26-29]. To confirm whether RSV-mediated maintenance of the self-renewal and multipotency of 6w-MSCs are due to the preservation of SIRT1-mediated SOX2 expression, we performed immunocytochemistry against SIRT1 and SOX2 during the proliferation and differentiation of 6w-MSCs. The 6w-MSCs were maintained in vehicle- or RSV-containing basal growth medium for 10 days, and then immunostaining against SIRT1 and SOX2 was performed to compare the levels of both proteins. Our results showed that in 6w-MSCs grown with vehicle-containing medium, the levels of SIRT1 and SOX2 were much weaker than in those grown with RSV-containing medium on Day 1, and both proteins were almost not detected from Day 4 to Day 10. In 6w-MSCs grown with RSV-containing medium, the levels of SIRT1 and SOX2 were much higher than those grown with vehicle-containing medium on Day 1, and although the levels gradually decreased over 10 days, both protein levels were still maintained from Day 4 to Day 10 (Fig. 4A). Likewise, the levels of SIRT1 and SOX2 were maintained higher during osteogenic and adipogenic differentiation when 6w-MSCs were cultured in RSV-containing differentiation media (Fig. 4B, 4C). These results indicate that MSCs with high differentiation potential can be obtained for clinical purposes, since RSV treatment allows MSCs to maintain high levels of SIRT1 and SOX2 during long-term *in vitro* cultivation.

**Figure 5.**
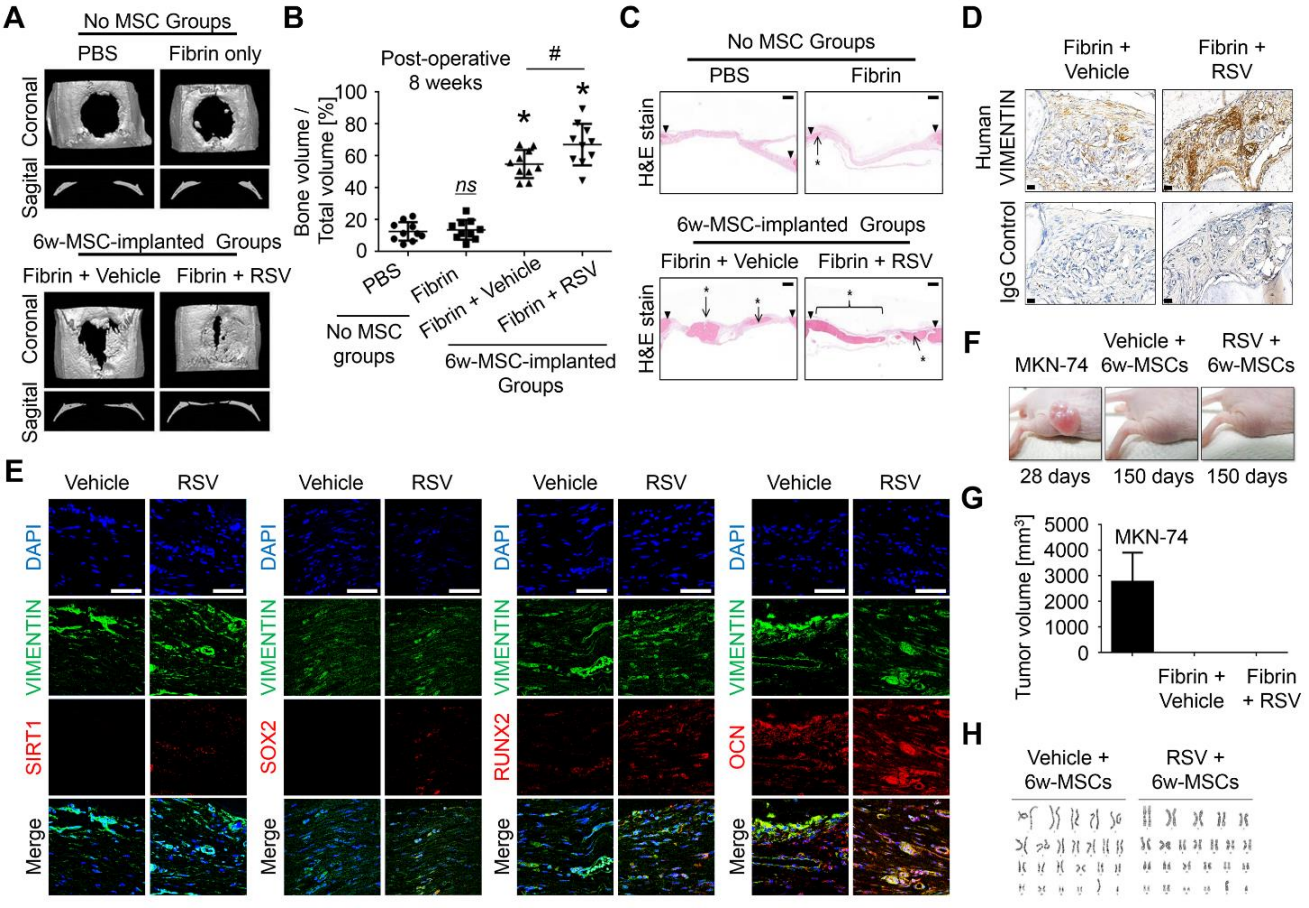
RSV induction improves bone healing potential of 6w-MSCs (**A**) Critical-sized calvarial defects (8-mm diameter) in rats were covered with fibrin glue, except for defect control treatment. Eight weeks after implantation, bone regeneration was measured by micro-computed tomography. A representative image is shown. (**B**) This graph shows the bone volume per mm^3^ (right panel) (*n* = 10). *, *p* < 0.05 compared to defect. #, *p* < 0.05 compared to 6w-MSCs treated with vehicle. (**C**) Hematoxylin and eosin staining was performed to observe new bone formation. The arrows show the edges of the host bone and line with asterisks indicates newly regenerated bone. Scale bar = 500 μm. (**D**) To confirm whether the newly regenerated bone was derived from a human origin, immunohistochemistry was performed using antibodies specific to human vimentin. The arrows indicate tissue derived from a human origin. Scale bar = 20 μm. (**E**) To confirm whether the transplanted 6w-MSCs contributed to bone regeneration of calvarial defects, immunohistochemistry was performed using antibodies against SIRT1, SOX2, RUNX2, and OCN as well as antibodies specific to human vimentin. The nucleus was stained with DAPI, and human VIMENTIN was stained with FITC-conjugated secondary antibody. SIRT1, SOX2, RUNX2, and OCN were stained with phycoerythrin (PE, red)-conjugated secondary antibody. Scale bar = 50 μm. (**F**) Effect of 6w-MSCs with RSV induction on tumorigenicity in 5-week-old female BALB/C nude mice. (**G**) Effect on the growth of MKN-74 cells, 6w-MSCs with vehicle induction, and 6w-MSCs with RSV induction xenografted in nude mice, showing no tumor growth in both 6w-MSC groups. (**H**) G-banding chromosome karyotype from 6w-MSCs with vehicle or RSV induction for 6 weeks. 6w-MSCs with 12th to 14th passages were used for animal experiments.

### RSV induction enhances 6w-MSC-driven bone regeneration

To address the clinical potential of RSV in MSC-driven bone regeneration, we investigated the regenerative potential of 6w-MSCs transplanted in a rat calvarial defect model. Critical size calvarial defects (8 mm diameter) were created and covered with fibrin glue mixed with the cells. At 4 weeks after implantation, micro-computed tomography analysis showed that bone regeneration was slightly increased in both 6w-MSCs with or without RSV induction (Supplementary Fig. 3). Eight weeks post-surgery, 6w-MSCs with RSV induction showed dramatically enhanced bone regeneration displayed by a better bone volume filled with mineralized bone compared to vehicle-treated MSCs (Fig. 5A, 5B). At 8 weeks post-transplantation, the bone bridge nearly filled with the defect in 6w-MSCs with RSV induction, whereas partially regenerated bone was observed in 6w-MSCs treated with vehicle (Fig. 5C). Immune response by allogenic cell transplantation to the recipient can be an important issue in clinical trials. Bone marrow-derived MSCs are known to have immune suppressive effects. Some reports have shown that MSCs suppress alloreactive T cells depending on cell doses [30], and do not elicit lymphocyte proliferation response [31]. In addition, MSCs can modulate immune responses at multiple levels by inhibiting the generation and function of monocyte-derived dendritic cells [32]. Recent studies showed no obvious immune response and inflammatory cells in animal tissues grafted with human MSCs [33, 34], thereby inducing successful tissue regeneration by MSCs without inflammation. In the current study, we also did not observe immune rejection responses, such as macrophage production, in rat calvarial tissues xenografted by human 6w-MSCs. Our results are in line with those of previous studies, indicating that MSCs have immune-modulatory effects in xenograft models. To confirm whether newly regenerated bone is derived from MSCs implanted *in vivo*, we performed immune-histochemistry using an antibody against human-specific vimentin, a marker of mesenchymal-derived cells because transplanted MSCs were of human origin[35]. Positive staining for human vimentin revealed that newly regenerated bone originated in implanted human MSCs (Fig. 5D). To explain this discrepancy on why 6w-MSCs treated with RSV had a greater bone regenerative property than that of control 6w-MSCs, we performed immunostaining against each SIRT1, SOX2, RUNX2, and OSTEOCALCIN (OCN), and they were cross-stained with human VIMENIN. RUNX2 is a major transcription factor of osteogenic differentiation, and OCN plays an essential role for the maturation of mineral formation [36]. Our results showed that in 6w-MSCs treated with RSV, human VIMENTIN-positive cells still retained SIRT1 and SOX2. In addition, levels of RUNX2 and OCN in RSV-treated 6w-MSC group were higher than those of control group and were positive to human VIMENTIN (Fig. 5E). Human VIMENTIN was not detected in groups with no human MSCs (supplementary Fig. 4). These results indicate that implanted human 6w-MSCs directly contribute to bone regeneration. To test the safety of long-term culture 6w-MSCs with RSV induction for MSC therapy, we analyzed the surface markers of MSCs, identified by the International Society for Cell Therapy [37], using flow cytometry in the absence or presence of RSV. All 6w-MSCs stably expressed CD90 and CD105, which are positive surface markers of MSCs, whereas the cells were negative for CD34 and CD45 (Supplementary Fig. 5), indicating that RSV induction did not alter the surface antigen profiles of MSCs. For *in vivo* tumorigenicity, 6w-MSCs with or without RSV induction were subcutaneously implanted into nude mice. We also injected MKN-74 cells, which are gastric adenocarcinoma cells, into nude mice as a positive control. As a result, MKN-74 adenocarcinoma cells formed tumors with a volume of 2.8 ± 1.2 cm^3^ by 28 days, whereas 6w-MSCs with or without RSV induction did not form visible tumors after 150 days (Fig. 5F, 5G). In the field of stem cell-based regenerative medicine, genetic instability that appears in stem cells during *ex vivo* manipulation such as *in vitro* expansion, culture condition, and transgenics [38], thus resulting in tumorigenesis after* in vivo* implantation of stem cells, is a major limitation to application of this method [39]. Therefore, we tested the chromosomal instability of MSCs in a G-banding karyotyping assay to analyze chromosomal aberrations. Both 6w-MSCs contained the first 22 pairs of chromosomes, known as autosomes, and sex chromosomes (XY). Additionally, there were no structural abnormalities in these chromosomes (Fig. 5F). These results indicate that RSV treatment does not lead to chromosome instability and tumor formation in MSCs, indicating that 6w-MSCs with RSV induction are safe for clinical application. In conclusion, we demonstrated the possibility of using 6w-MSCs with RSV induction for stem cell therapy which showed sufficient efficacy and safety for clinical application. There are several potential benefits to using RSV as an additive to MSC cultures, including the faster acquisition of a sufficient number of cells, easy treatment procedure, high efficacy of differentiation, and no genotoxicity. This method overcomes the major limitations to clinical application of MSCs in tissue regeneration.

## DISCUSSION

MSCs are used for skeletal tissue engineering, but they typically lose their stem cell characteristics during *in vitro* expansion, which is a necessary step to obtain a sufficient number of cells for disease treatment [6, 7, 40]. For the development of stem cell therapy, it is important for MSCs to maintain their stem cell features during long-term *in vitro* expansion. In addition, genotoxicity must be considered because it is possible for stem cells maintained *in vitro* to generate tumors when they are transplanted *in vivo* [41]. Herein, we report the possibility that MSCs expanded in culture with RSV can be used for stem cell therapy and have sufficient efficacy and safety for clinical application. There are several potential benefits to using RSV as an additive for MSC cultures, including the faster acquisition of a sufficient number of cells, an easy treatment procedure, a high efficacy of differentiation, and no genotoxicity. These results can eliminate major obstacles for the clinical application of MSCs in tissue regeneration. RSV is a natural polyphenol compound that is well-known as a potent SIRT1 activator and has been reported to have beneficial effects against aging and various human diseases including cancer [41, 42]. Our previous studies revealed that SIRT1, which functions as an NAD^+^-dependent lysine deacetylase, prevents SOX2 against degradation by deacetylating it, thus maintaining the stemness of MSCs after extended passaging [16]. Indeed, RSV could retain the self-renewal and multi-lineage differentiation capacities of human MSCs beyond passage 10 through a SIRT1-dependent pathway [12, 16]. Additionally, SIRT1 indirectly regulates the expression of *OCT4* and *NANOG*, which is normally suppressed by P53, by deacetylating P53 in human embryonic stem cells and cancer stem cells [43, 44], while RSV was reported to reduce the protein level of P53 through inducing its deacetylation in adult stem cells [45]. In agreement with these reports, Figure 3b showed that the protein level of SOX2 and NANOG were almost entirely retained in RSV-treated MSCs. However, the protein of OCT4 did not significantly change. Previous studies have reported that OCT4 is dispensable for the maintenance of the self-renewal ability in somatic cells [46] and is independent on maintaining stemness of MSCs during their *in vitro* expansion [29], suggesting that OCT4 is less likely to be regulated during the long-term passaging of MSCs in culture. Based on these results, RSV treatment can retain the protein abundance of SOX2 through a SIRT1-dependent pathway and can also retain that of NANOG via the regulation of P53, but it does not affect the decline in the abundance of OCT4 during long-term passaging.

Although it is a well-known SIRT1 activator, RSV also regulates diverse other proteins and signaling pathways in addition to SIRT1 [47]. In adipocytes, RSV modulates the PI3K/AKT and MAPK/ERK pathways in a SIRT1-independent manner, thus reducing the number and lipid accumulation of the adipocytes [48]. In addition, RSV was reported to induce cancer cell death via the ERK1/2-P53 axis [49]. It has been reported that the PI3K/AKT and ERK signaling pathways upregulate *RUNX2* expression, finally resulting in tooth development and regeneration [50], which means that these signaling pathways are also involved in bone regeneration. In other words, the enhanced bone regeneration capacity of 6w-MSCs with RSV induction could be associated with the regulation of other proteins and pathways by RSV in addition to its effect on SIRT1. In the present study, we did not confirm whether RSV treatment regulates other pathways such as PI3K, AKT, and ERK during the osteogenic differentiation of MSCs. However, our previous study revealed that the osteogenic differentiation potential of MSCs treated with RSV was abolished in SIRT1-deficient cells [12]. A recent study suggested that RSV stabilizes SIRT1/peptide interactions in a substrate-specific manner [51], which means that RSV can directly bind to SIRT1 protein. In a previous study, we demonstrated that SIRT1 physically interacts with SOX2, and the direct interaction stabilizes SOX2 protein by deacetylating lysine residues in order to block the nuclear export and ubiquitination of SOX2 protein [16]. Collectively, RSV-activated signaling pathway is shown as direct interaction of RSV-SIRT1-SOX2 complex. In current study, we showed that RSV could not enhance the proliferation and differentiation potentials of MSCs without SIRT1 (Fig. 3G-3K). Likewise, our previous study also showed that RSV cannot enhance the proliferation and differentiation potentials of MSCs without SOX2 [16]; therefore, RSV-mediated enhancement of MSC function is shown to be directly involved with SIRT1-SOX2. In this study, we also confirmed the effect of RSV on maintaining multi-lineage differentiation potential of MSCs is mediated by SIRT1-SOX2 axis, suggesting that the effect of RSV on bone regeneration may rely on the presence of SIRT1.

The relationship between self-renewal, osteogenic, and adipogenic capacities of MSCs has been found to be antagonistic and competitive in various MSC-related studies. For this reason, many researchers may believe that enhancing the self-renewal capacity by some regulators or molecules enables suppression of stem cell differentiation, which means that undifferentiated state (no loss of differentiation potentials) of stem cells can be achieved by maintaining the self-renewal capacity of stem cells. Since MSC osteogenesis and adipogenesis are also shown to be competitive in differentiation condition, it can be thought that enhancing the osteogenic capacity of MSCs by some regulators or molecules enables suppression of the adipogenic potential of MSCs. However, various factors or proteins may be involved in the RSV-mediated enhancement of MSC self-renewal and differentiation potentials. RSV can mainly activate SIRT1 to maintain the self-renewal and differentiation capacities in human MSCs [16]. The SIRT1 activated by RSV can affect MSC self-renewal-related regulators, such as SOX2 [16] and FOXO3A [52], and MSC osteogenesis-related regulators, such as RUNX2 [53] and Wnt/β-catenin [54]. Some studies suggested that RSV decreased adipocyte differentiation and lipid accumulation in 3T3-L1 cells preadipocytes [55, 56], resulting in loss of body weight [57]. Collectively, it has been shown that RSV positively regulates MSC self-renewal and osteogenesis while negatively regulating MSC adipogenesis. However, the current study showed that RSV enhanced MSC adipogenic potential as well as the self-renewal and osteogenic potentials. These results can be explained by the fact that RSV-activated SIRT1 works in the cells by removing acetyl groups from protein substrates related to aging processes of MSCs in order to delay or prevent cellular aging. Delaying or preventing MSC aging by applying RSV *in vitro* enables gathering of healthy MSCs, which have self-renewal and multi-differentiation potentials during long-term cultivation. This means that *in vitro* RSV treatment maintains healthy MSCs over long-term cultivation, thereby resulting in better proliferation and differentiation potentials compared to the cells cultured without RSV. It can also be thought that RSV suppresses *in vivo* adipogenesis of MSCs if proper materials with RSV can be applied into *in vivo* models; however, we could not confirm the *in vivo* effect of RSV on MSC adipogenesis as this study aimed to confirm whether RSV-treated MSCs had higher therapeutic potential when cells were transplanted into *in vivo* bone defect model. In this study, RSV effect *in vivo* refers to the bone regenerative capacity of MSCs pre-treated with RSV for long-term duration (6w-MSCs) in a rat calvarial defect model. We wanted to confirm whether bone regenerative potential of MSCs after the *in vitro* long-term cultivation for obtaining MSCs with effective cell doses could be preserved when the cells were cultured in RSV-containing culture medium. It is important to gather the number of cells with effective dosage for MSC therapy; however, after passaging during long-term culture, most of the bone marrow-derived MSCs lose their potential to differentiate into multiple lineages as well as self-renewal capacity. Although RSV treatment is known to activate MSC proliferative and differentiation potentials, there is still not enough evidence to confirm whether RSV-treated MSCs are indeed effective in tissue regeneration when transplanted into *in vivo* disease models. Although RSV was treated with MSC cultures in this study, we believe it is possible for RSV to be injected into some disease models if appropriate materials are available for bone defects. This study provides a possibility that natural small molecules, such as RSV, can be employed in MSC culture in order to enhance therapeutic potential of cells. Therefore, we suggest that using small molecules would offer more benefits than using growth factors to enhance MSC stemness.

Comprehensively, our study demonstrates that RSV treatment can enhance proliferation of MSCs and maintain differentiation potential during long-term expansion *in vitro*, and transplantation of MSCs pre-treated with RSV improves bone regeneration in rat calvarial defect model. Although we transplanted MSCs mixed with fibrin glue for MSC delivery *in vivo*, examining alternative materials to provide the best environments for cell adherence, differentiation into cartilage, and effective delivery may facilitate more effective tissue regeneration.

## Supplemental data

Supplemental data are available at www.aginganddisease.org/EN/10.14336/AD.2018.0802.



## References

[1] Pittenger MF, Mackay AM, Beck SC, Jaiswal RK, Douglas R, Mosca JD, et al (1999). Multilineage potential of adult human mesenchymal stem cells. Science, 284: 143-147. 10102814 10.1126/science.284.5411.143

[2] Niyibizi C, Li F (2009). Potential implications of cell therapy for osteogenesis imperfecta. Int J Clin Rheumtol, 4: 57-66. 20490372 10.2217/17584272.4.1.57 PMC2873227

[3] Chan JK, Gotherstrom C (2014). Prenatal transplantation of mesenchymal stem cells to treat osteogenesis imperfecta. Front Pharmacol, 5: 223. 25346689 10.3389/fphar.2014.00223 PMC4191163

[4] Gupta PK, Das AK, Chullikana A, Majumdar AS (2012). Mesenchymal stem cells for cartilage repair in osteoarthritis. Stem Cell Res Ther, 3: 25. 22776206 10.1186/scrt116 PMC3580463

[5] Burke J, Hunter M, Kolhe R, Isales C, Hamrick M, Fulzele S (2016). Therapeutic potential of mesenchymal stem cell based therapy for osteoarthritis. Clin Transl Med, 5: 27. 27510262 10.1186/s40169-016-0112-7 PMC4980326

[6] Rombouts WJ, Ploemacher RE (2003). Primary murine MSC show highly efficient homing to the bone marrow but lose homing ability following culture. Leukemia, 17: 160-170. 12529674 10.1038/sj.leu.2402763

[7] Ksiazek K (2009). A comprehensive review on mesenchymal stem cell growth and senescence. Rejuvenation Res, 12: 105-116. 19405814 10.1089/rej.2009.0830

[8] Bellantuono I, Aldahmash A, Kassem M (2009). Aging of marrow stromal (skeletal) stem cells and their contribution to age-related bone loss. Biochim Biophys Acta, 1792: 364-370. 19419706 10.1016/j.bbadis.2009.01.008

[9] Howitz KT, Bitterman KJ, Cohen HY, Lamming DW, Lavu S, Wood JG, et al (2003). Small molecule activators of sirtuins extend Saccharomyces cerevisiae lifespan. Nature, 425: 191-196. 12939617 10.1038/nature01960

[10] Rahman I (2008). Dietary polyphenols mediated regulation of oxidative stress and chromatin remodeling in inflammation. Nutr Rev, 66 Suppl 1: S42-45. 18673489 10.1111/j.1753-4887.2008.00067.x PMC2556856

[11] Dai Z, Li Y, Quarles LD, Song T, Pan W, Zhou H, et al (2007). Resveratrol enhances proliferation and osteoblastic differentiation in human mesenchymal stem cells via ER-dependent ERK1/2 activation. Phytomedicine, 14: 806-814. 17689939 10.1016/j.phymed.2007.04.003

[12] Yoon DS, Choi Y, Choi SM, Park KH, Lee JW (2015). Different effects of resveratrol on early and late passage mesenchymal stem cells through beta-catenin regulation. Biochem Biophys Res Commun, 467: 1026-1032. 26456654 10.1016/j.bbrc.2015.10.017

[13] Peltz L, Gomez J, Marquez M, Alencastro F, Atashpanjeh N, Quang T, et al (2012). Resveratrol exerts dosage and duration dependent effect on human mesenchymal stem cell development. PLoS One, 7: e37162. 22615926 10.1371/journal.pone.0037162 PMC3353901

[14] Yuan HF, Zhai C, Yan XL, Zhao DD, Wang JX, Zeng Q, et al (2012). SIRT1 is required for long-term growth of human mesenchymal stem cells. J Mol Med (Berl), 90: 389-400. 22038097 10.1007/s00109-011-0825-4

[15] Fischer-Posovszky P, Kukulus V, Tews D, Unterkircher T, Debatin KM, Fulda S, et al (2010). Resveratrol regulates human adipocyte number and function in a Sirt1-dependent manner. Am J Clin Nutr, 92: 5-15. 20463039 10.3945/ajcn.2009.28435

[16] Yoon DS, Choi Y, Jang Y, Lee M, Choi WJ, Kim SH, et al (2014). SIRT1 directly regulates SOX2 to maintain self-renewal and multipotency in bone marrow-derived mesenchymal stem cells. Stem Cells, 32: 3219-3231. 25132403 10.1002/stem.1811

[17] Yoon DS, Kim YH, Lee S, Lee KM, Park KH, Jang Y, et al (2014). Interleukin-6 induces the lineage commitment of bone marrow-derived mesenchymal multipotent cells through down-regulation of Sox2 by osteogenic transcription factors. FASEB J, 28: 3273-3286. 24719354 10.1096/fj.13-248567

[18] Yoon DS, Lee KM, Kim SH, Jung Y, Park KH, Choi Y, et al (2016). Synergistic Action of IL-8 and Bone Marrow Concentrate on Cartilage Regeneration Through Upregulation of Chondrogenic Transcription Factors. Tissue Eng Part A, 22: 363-374. 26871861 10.1089/ten.tea.2015.0425

[19] Park JS, Chang DY, Kim JH, Jung JH, Park J, Kim SH, et al (2013). Retrovirus-mediated transduction of a cytosine deaminase gene preserves the stemness of mesenchymal stem cells. Exp Mol Med, 45: e10. 23429359 10.1038/emm.2013.21 PMC3584665

[20] Oryan A, Kamali A, Moshiri A, Baghaban Eslaminejad M (2017). Role of Mesenchymal Stem Cells in Bone Regenerative Medicine: What Is the Evidence? Cells Tissues Organs, 204: 59-83. 28647733 10.1159/000469704

[21] Bertoli C, Skotheim JM, de Bruin RA (2013). Control of cell cycle transcription during G1 and S phases. Nat Rev Mol Cell Biol, 14: 518-528. 23877564 10.1038/nrm3629 PMC4569015

[22] Duronio RJ, Xiong Y (2013). Signaling pathways that control cell proliferation. Cold Spring Harb Perspect Biol, 5: a008904. 23457258 10.1101/cshperspect.a008904 PMC3578363

[23] Hua P, Liu J, Tao J, Yang S (2015). Influence of caspase-3 silencing on the proliferation and apoptosis of rat bone marrow mesenchymal stem cells under hypoxia. Int J Clin Exp Med, 8: 1624-1633. 25932091 PMC4402738

[24] Turinetto V, Vitale E, Giachino C (2016). Senescence in Human Mesenchymal Stem Cells: Functional Changes and Implications in Stem Cell-Based Therapy. Int J Mol Sci, 17. 10.3390/ijms17071164 PMC4964536 27447618

[25] Mu WL, Wang YJ, Xu P, Hao DL, Liu XZ, Wang TT, et al (2015). Sox2 Deacetylation by Sirt1 Is Involved in Mouse Somatic Reprogramming. Stem Cells, 33: 2135-2147. 25940188 10.1002/stem.2012

[26] Han SM, Han SH, Coh YR, Jang G, Chan Ra J, Kang SK, et al (2014). Enhanced proliferation and differentiation of Oct4- and Sox2-overexpressing human adipose tissue mesenchymal stem cells. Exp Mol Med, 46: e101. 24946789 10.1038/emm.2014.28 PMC4081551

[27] Fan YX, Gu CH, Zhang YL, Zhong BS, Wang LZ, Zhou ZR, et al (2013). Oct4 and Sox2 overexpression improves the proliferation and differentiation of bone mesenchymal stem cells in Xiaomeishan porcine. Genet Mol Res, 12: 6067-6079. 24338401 10.4238/2013.December.2.5

[28] Park SB, Seo KW, So AY, Seo MS, Yu KR, Kang SK, et al (2012). SOX2 has a crucial role in the lineage determination and proliferation of mesenchymal stem cells through Dickkopf-1 and c-MYC. Cell Death Differ, 19: 534-545. 22015605 10.1038/cdd.2011.137 PMC3278737

[29] Yoon DS, Kim YH, Jung HS, Paik S, Lee JW (2011). Importance of Sox2 in maintenance of cell proliferation and multipotency of mesenchymal stem cells in low-density culture. Cell Prolif, 44: 428-440. 21951286 10.1111/j.1365-2184.2011.00770.x PMC6495637

[30] Le Blanc K, Tammik L, Sundberg B, Haynesworth SE, Ringden O (2003). Mesenchymal stem cells inhibit and stimulate mixed lymphocyte cultures and mitogenic responses independently of the major histocompatibility complex. Scand J Immunol, 57: 11-20. 12542793 10.1046/j.1365-3083.2003.01176.x

[31] Le Blanc K, Tammik C, Rosendahl K, Zetterberg E, Ringden O (2003). HLA expression and immunologic properties of differentiated and undifferentiated mesenchymal stem cells. Exp Hematol, 31: 890-896. 14550804 10.1016/s0301-472x(03)00110-3

[32] Nauta AJ, Kruisselbrink AB, Lurvink E, Willemze R, Fibbe WE (2006). Mesenchymal stem cells inhibit generation and function of both CD34+-derived and monocyte-derived dendritic cells. J Immunol, 177: 2080-2087. 16887966 10.4049/jimmunol.177.4.2080

[33] Zang S, Zhu L, Luo K, Mu R, Chen F, Wei X, et al (2017). Chitosan composite scaffold combined with bone marrow-derived mesenchymal stem cells for bone regeneration: in vitro and in vivo evaluation. Oncotarget, 8: 110890-110903. 29340024 10.18632/oncotarget.22917 PMC5762292

[34] Takeshita K, Motoike S, Kajiya M, Komatsu N, Takewaki M, Ouhara K, et al (2017). Xenotransplantation of interferon-gamma-pretreated clumps of a human mesenchymal stem cell/extracellular matrix complex induces mouse calvarial bone regeneration. Stem Cell Res Ther, 8: 101. 28446226 10.1186/s13287-017-0550-1 PMC5406942

[35] Jafari A, Qanie D, Andersen TL, Zhang Y, Chen L, Postert B, et al (2017). Legumain Regulates Differentiation Fate of Human Bone Marrow Stromal Cells and Is Altered in Postmenopausal Osteoporosis. Stem Cell Reports, 8: 373-386. 28162997 10.1016/j.stemcr.2017.01.003 PMC5312427

[36] Komori T (2003). Requisite roles of Runx2 and Cbfb in skeletal development. J Bone Miner Metab, 21: 193-197. 12811622 10.1007/s00774-002-0408-0

[37] Dominici M, Le Blanc K, Mueller I, Slaper-Cortenbach I, Marini F, Krause D, et al (2006). Minimal criteria for defining multipotent mesenchymal stromal cells. The International Society for Cellular Therapy position statement. Cytotherapy, 8: 315-317. 16923606 10.1080/14653240600855905

[38] Knoepfler PS (2009). Deconstructing stem cell tumorigenicity: a roadmap to safe regenerative medicine. Stem Cells, 27: 1050-1056. 19415771 10.1002/stem.37 PMC2733374

[39] Lee AS, Tang C, Rao MS, Weissman IL, Wu JC (2013). Tumorigenicity as a clinical hurdle for pluripotent stem cell therapies. Nat Med, 19: 998-1004. 23921754 10.1038/nm.3267 PMC3967018

[40] Bruder SP, Jaiswal N, Haynesworth SE (1997). Growth kinetics, self-renewal, and the osteogenic potential of purified human mesenchymal stem cells during extensive subcultivation and following cryopreservation. J Cell Biochem, 64: 278-294. 9027588 10.1002/(sici)1097-4644(199702)64:2<278::aid-jcb11>3.0.co;2-f

[41] Valenzano DR, Terzibasi E, Genade T, Cattaneo A, Domenici L, Cellerino A (2006). Resveratrol prolongs lifespan and retards the onset of age-related markers in a short-lived vertebrate. Curr Biol, 16: 296-300. 16461283 10.1016/j.cub.2005.12.038

[42] Signorelli P, Ghidoni R (2005). Resveratrol as an anticancer nutrient: molecular basis, open questions and promises. J Nutr Biochem, 16: 449-466. 16043028 10.1016/j.jnutbio.2005.01.017

[43] Zhang ZN, Chung SK, Xu Z, Xu Y (2014). Oct4 maintains the pluripotency of human embryonic stem cells by inactivating p53 through Sirt1-mediated deacetylation. Stem Cells, 32: 157-165. 24038750 10.1002/stem.1532 PMC3947311

[44] Chen X, Sun K, Jiao S, Cai N, Zhao X, Zou H, et al (2014). High levels of SIRT1 expression enhance tumorigenesis and associate with a poor prognosis of colorectal carcinoma patients. Sci Rep, 4: 7481. 25500546 10.1038/srep07481 PMC4265776

[45] Liu B, Ghosh S, Yang X, Zheng H, Liu X, Wang Z, et al (2012). Resveratrol rescues SIRT1-dependent adult stem cell decline and alleviates progeroid features in laminopathy-based progeria. Cell Metab, 16: 738-750. 23217256 10.1016/j.cmet.2012.11.007

[46] Lengner CJ, Camargo FD, Hochedlinger K, Welstead GG, Zaidi S, Gokhale S, et al (2007). Oct4 expression is not required for mouse somatic stem cell self-renewal. Cell Stem Cell, 1: 403-415. 18159219 10.1016/j.stem.2007.07.020 PMC2151746

[47] Baur JA, Sinclair DA (2006). Therapeutic potential of resveratrol: the in vivo evidence. Nat Rev Drug Discov, 5: 493-506. 16732220 10.1038/nrd2060

[48] Baile CA, Yang JY, Rayalam S, Hartzell DL, Lai CY, Andersen C, et al (2011). Effect of resveratrol on fat mobilization. Ann N Y Acad Sci, 1215: 40-47. 21261640 10.1111/j.1749-6632.2010.05845.x

[49] Lin HY, Lansing L, Merillon JM, Davis FB, Tang HY, Shih A, et al (2006). Integrin alphaVbeta3 contains a receptor site for resveratrol. Faseb j, 20: 1742-1744. 16790523 10.1096/fj.06-5743fje

[50] Someya H, Fujiwara H, Nagata K, Wada H, Hasegawa K, Mikami Y, et al (2015). Thymosin beta 4 is associated with RUNX2 expression through the Smad and Akt signaling pathways in mouse dental epithelial cells. Int J Mol Med, 35: 1169-1178. 25739055 10.3892/ijmm.2015.2118 PMC4380193

[51] Hou X, Rooklin D, Fang H, Zhang Y (2016). Resveratrol serves as a protein-substrate interaction stabilizer in human SIRT1 activation. Sci Rep, 6: 38186. 27901083 10.1038/srep38186 PMC5128864

[52] Tseng PC, Hou SM, Chen RJ, Peng HW, Hsieh CF, Kuo ML, et al (2011). Resveratrol promotes osteogenesis of human mesenchymal stem cells by upregulating RUNX2 gene expression via the SIRT1/FOXO3A axis. J Bone Miner Res, 26: 2552-2563. 21713995 10.1002/jbmr.460

[53] Shakibaei M, Shayan P, Busch F, Aldinger C, Buhrmann C, Lueders C, et al (2012). Resveratrol mediated modulation of Sirt-1/Runx2 promotes osteogenic differentiation of mesenchymal stem cells: potential role of Runx2 deacetylation. PLoS One, 7: e35712. 22539994 10.1371/journal.pone.0035712 PMC3335081

[54] Gao X, Ge J, Li W, Zhou W, Xu L (2018). LncRNA KCNQ1OT1 promotes osteogenic differentiation to relieve osteolysis via Wnt/beta-catenin activation. Cell Biosci, 8: 19. 29541443 10.1186/s13578-018-0216-4 PMC5842584

[55] Rayalam S, Yang JY, Ambati S, Della-Fera MA, Baile CA (2008). Resveratrol induces apoptosis and inhibits adipogenesis in 3T3-L1 adipocytes. Phytother Res, 22: 1367-1371. 18688788 10.1002/ptr.2503

[56] Chen S, Li Z, Li W, Shan Z, Zhu W (2011). Resveratrol inhibits cell differentiation in 3T3-L1 adipocytes via activation of AMPK. Can J Physiol Pharmacol, 89: 793-799. 22017765 10.1139/y11-077

[57] Ahn J, Cho I, Kim S, Kwon D, Ha T (2008). Dietary resveratrol alters lipid metabolism-related gene expression of mice on an atherogenic diet. J Hepatol, 49: 1019-1028. 18930334 10.1016/j.jhep.2008.08.012

