# Supplementary material for: Enhancement of Mesenchymal Stem Cell-Driven Bone Regeneration by Resveratrol-Mediated SOX2 Regulation
Source: Aging Dis. 2019 Aug 1;10(4):818–33. doi: 10.14336/AD.2018.0802 (PMC6675538; doi:10.14336/AD.2018.0802)
Supplement: Supplementary file 1 [file AD-10-4-818-s.pdf]

SUPPLEMENTARY DATA

Original Article

Enhancement of Mesenchymal Stem Cell-Driven Bone Regeneration by Resveratrol-Mediated SOX2 Regulation

Yoorim Choi[1,2,#], Dong Suk Yoon[3,#], Kyoung-Mi Lee[1,4], Seong Mi Choi[1,2], Myon-Hee Lee[3,5], Kwang Hwan Park[1], Seung Hwan Han[6], Jin Woo Lee[1,2,4,*]

[1]Department of Orthopaedic Surgery, Yonsei University College of Medicine, Seoul 03722, South Korea. [2]Brain Korea 21 PLUS Project for Medical Science, Yonsei University College of Medicine, Seoul 03722, South Korea. [3]Department of Internal Medicine, Brody School of Medicine at East Carolina University, Greenville, North Carolina 27834, USA. [4]Severance Biomedical Science Institute, Yonsei University College of Medicine, 50-1 Yonsei -ro, Seodaemun-gu, Seoul 03722, South Korea. [5]Lineberger Comprehensive Cancer Center, University of North Carolina-Chapel Hill, Chapel Hill, North Carolina 27599, USA. [6]Department of Orthopaedic Surgery, Gangnam Severance Hospital, Yonsei University College of Medicine, Seoul 135-720, South Korea.

© 2018. Choi Y et al. Published online at http://www.aginganddisease.org/EN/10.14336/AD.2018.0802

SUPPLEMENTARY DATA

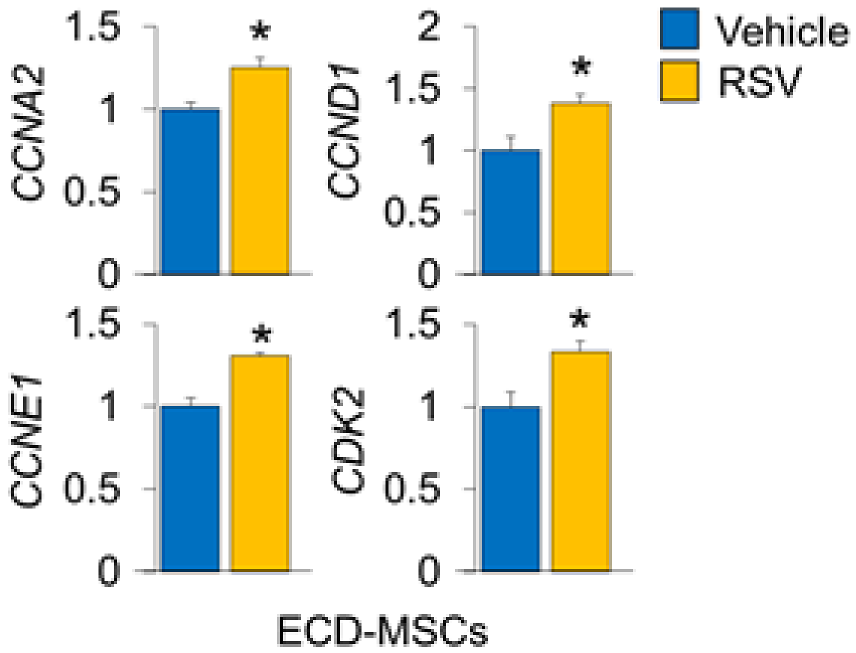

Supplementary Figure 1. Real-time quantitative PCR for cell cycle-related genes. The mRNA expression levels of *CCNA*, *CCND*, *CDK2*, and *CCNE* were analyzed by quantitative real-time polymerase chain reaction (qRT-PCR) in triplicate. *, $p < 0.05$.

© 2018. Choi Y et al. Published online at http://www.aginganddisease.org/EN/10.14336/AD.2018.0802

SUPPLEMENTARY DATA

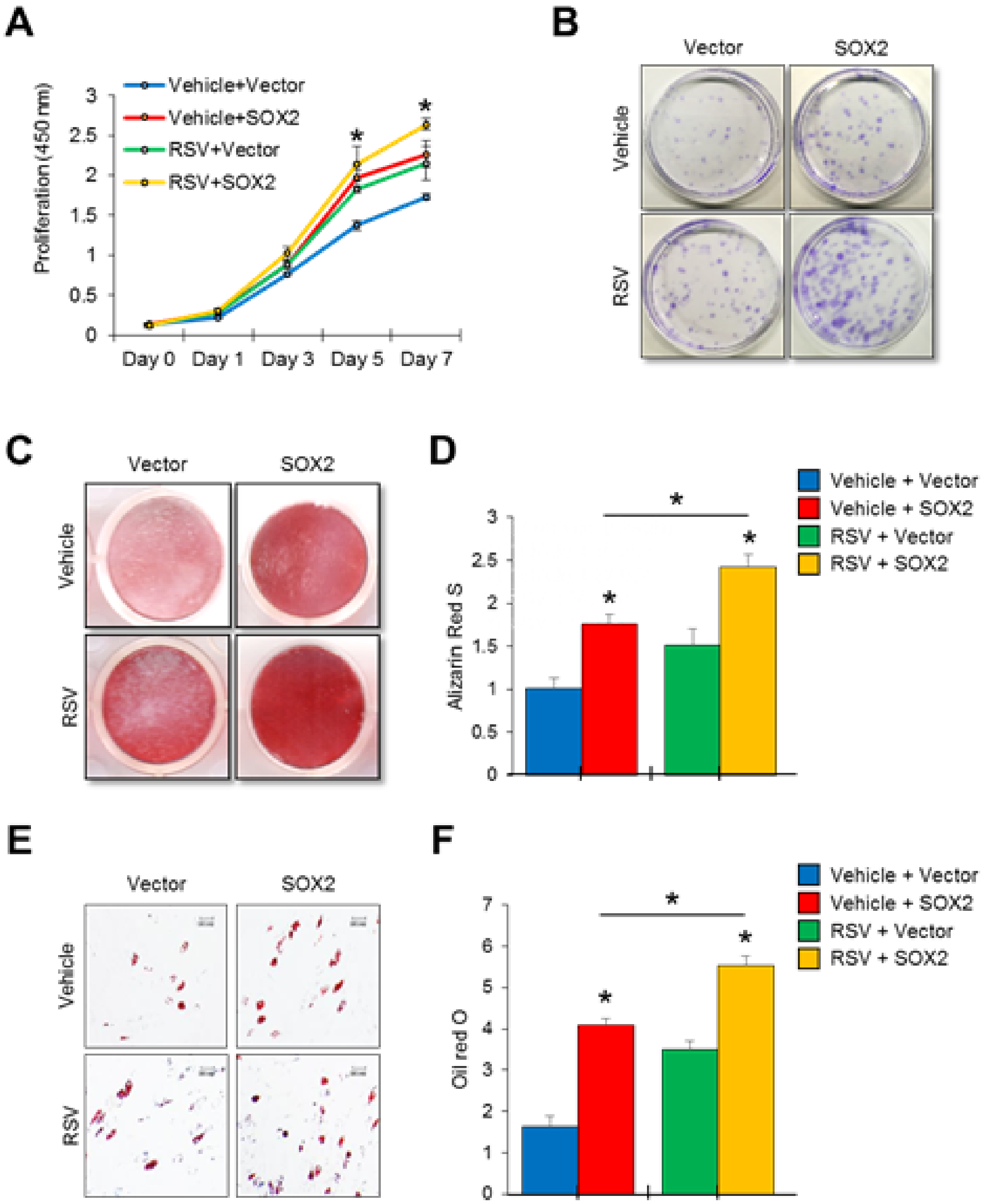

Supplementary Figure 2. SOX2 overexpression solely regulates the self-renewal and multi-potential activities of MSCs, but RSV treatment can improve the SOX2-mediated increase of MSC stemness. (**A**) A cell proliferation assay was performed to determine the proliferative capacities of 6w-MSCs transfected with vector control or SOX2 overexpression vector and treated with vehicle (0.05% EtOH) or 1 µM RSV using an EZ-Cytox Kit. Each experiment was performed in triplicate (n=3). (**B**) 6w-MSCs (1×10^3 cells per well in 100-mm dishes) transfected with vector control or SOX2 overexpression vector and treated with vehicle (0.05% EtOH) or 1 µM RSV were incubated in basal growth medium for 12 days. (**C**) 6w-MSCs (8×10^4 cells per well in 12-well plates) transfected with vector control or SOX2 overexpression vector and treated with vehicle (0.05% EtOH) or 1 µM RSV were incubated in osteogenic medium for 10 days. Alizarin red S staining was performed to detect mineral deposition at day 10. (**D**) For quantitative analysis, absorbance was measured at 595 nm following destaining with 10% cetylpyridinium for 30 min. *, $p < 0.05$ compared to vector control or vehicle. (**E**) 6w-MSCs (8×10^4 cells per well in 12-well plates) transfected with vector control or SOX2 overexpression vector and treated with vehicle (0.05% EtOH) or 1 µM RSV were incubated in adipogenic medium for 10 days. (**F**) For quantitative analysis, absorbance was measured at 500 nm following destaining with 100% isopropanol for 30 min. *, $p < 0.05$ compared to vector control or vehicle.

© 2018. Choi Y et al. Published online at http://www.aginganddisease.org/EN/10.14336/AD.2018.0802

SUPPLEMENTARY DATA

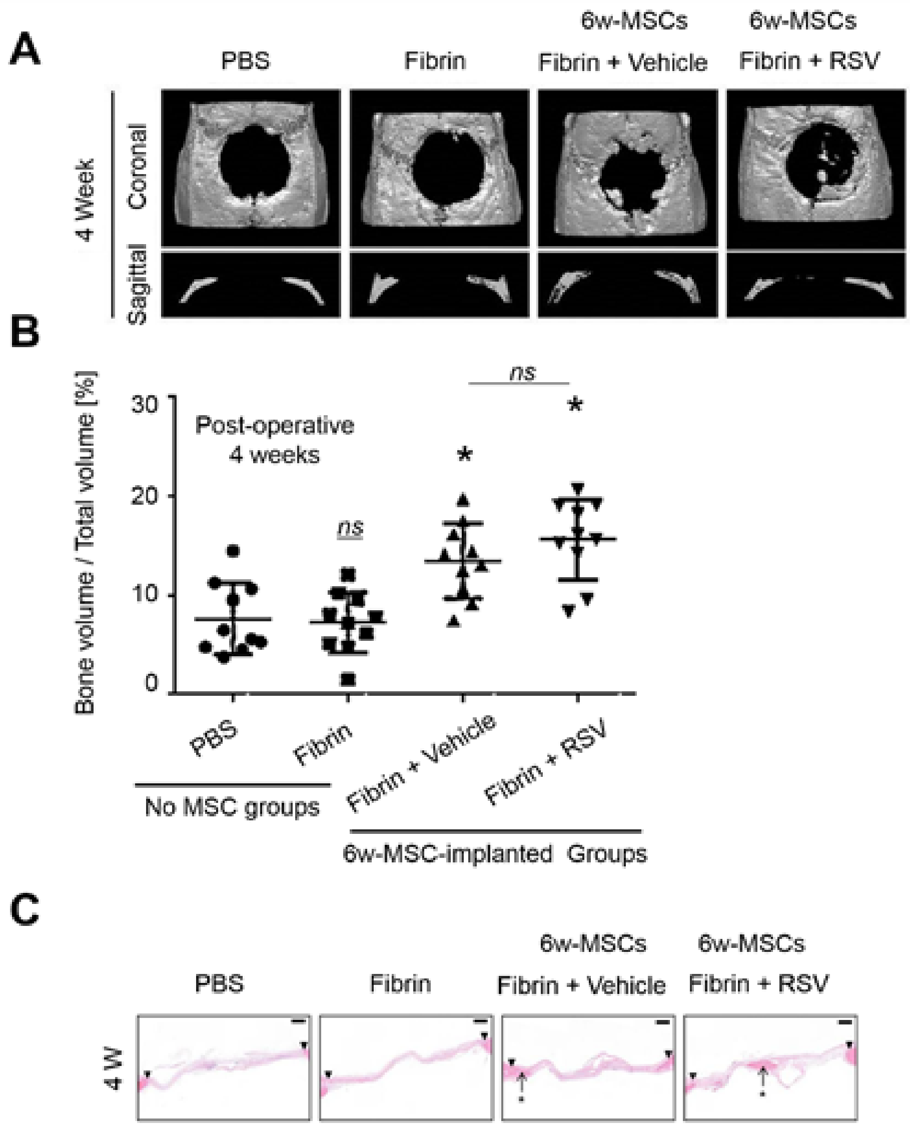

Supplementary Figure 3. The effect of ECD-MSCs on rat calvarial regeneration at 4 weeks post-operation. (**A**) Critical-sized calvarial defects (8-mm diameter) in rats were covered with a fibrin glue, except for the defect control treatment. Eight weeks after implantation, bone regeneration was measured by micro-computed tomography. A representative image is shown. (**B**) This graph shows the bone volume per mm^3 (right panel) (n = 10). *, $p < 0.05$ compared to defect. #, p < 0.05 compared to EDC-MSCs with vehicle. (**C**) Hematoxylin and eosin staining was performed to observe new bone formation. The arrows show the edges of the host bone and the line with asterisks indicates the newly regenerated bone. Scale bar = 500 μm.

© 2018. Choi Y et al. Published online at http://www.aginganddisease.org/EN/10.14336/AD.2018.0802

SUPPLEMENTARY DATA

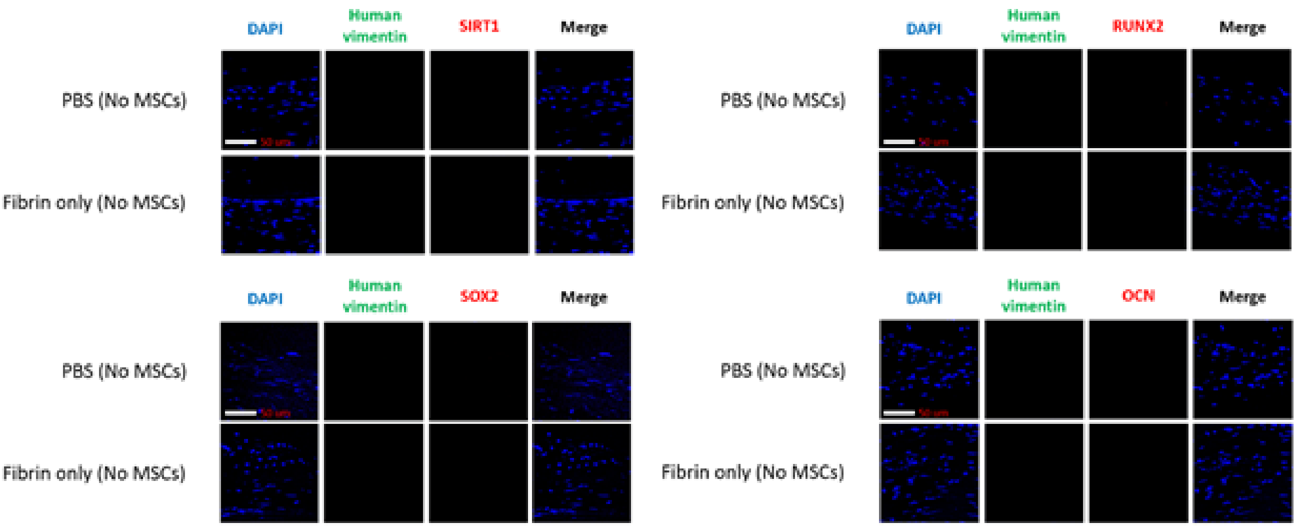

Supplementary Figure 4. Immunohistochemistry in the groups that human MSCs were not implanted into a rat calvarial defect. To confirm whether the transplanted 6w-MSCs contributed to bone regeneration of the calvarial defects, immunohistochemistry was performed using antibodies against SIRT1, SOX2, RUNX2, and OCN as well as antibodies specific to human vimentin. The nucleus was stained with DAPI and human VIMENTIN was stained with FITC-conjugated secondary antibody. SIRT1, SOX2, RUNX2, and OCN were stained with phycoerythrin (PE, red)-conjugated secondary antibody. Scale bar = 50 µm.

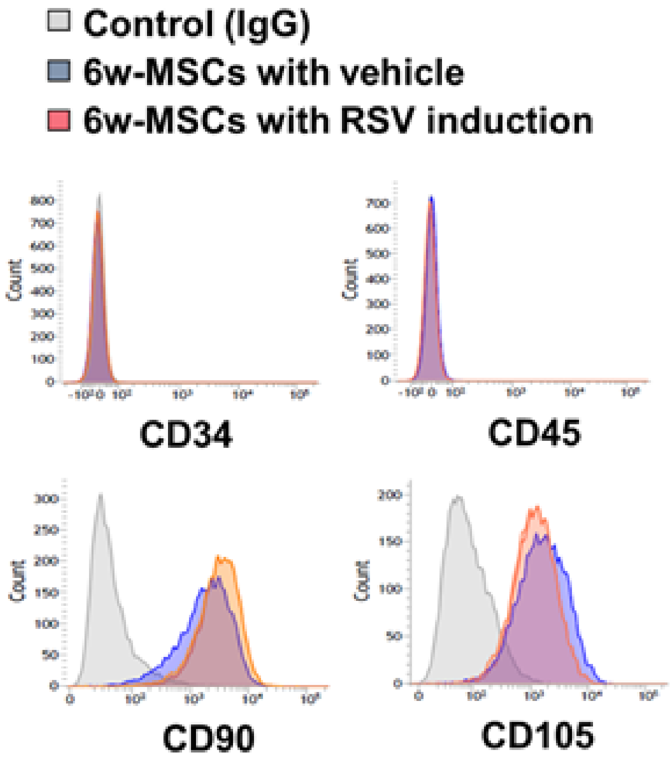

Supplementary Figure 5. No effect on the properties of MSCs in 6w-MSCs with or without RSV induction. ECD-MSCs with or without RSV Induction were stained with antibody conjugated with CD34-FITC, control IgG-FITC, CD45-PerCP, control IgG-PerCP, CD90-APC, control IgG-APC, CD105-PE-Cy7 and control IgG-PE-Cy7, and analyzed with flow cytometry.

© 2018. Choi Y et al. Published online at http://www.aginganddisease.org/EN/10.14336/AD.2018.0802

SUPPLEMENTARY DATA

Supplementary Table 1. Therapeutics Use of Mesenchymal Stem Cells (MSCs) in Skeletal Disease and Tissue Regeneration.

Disease	Cell type	Therapeutic dose	Days from Culture	Outcome	Refs.
**Cartilage defect**	BMSCs	5×10^{6} cells	28 days	Improved but subchondral irregularities emerged	[1]
	ADSCs	1×10^{8} cells	-	Improved	[2]
**Osteoarthritis (OA)**	ADSCs	3.9×10^{6} cells	-	Not significant	[3]
	BSMCs	8×10^{6} cells	28 - 35 days	Slightly improved	[4]
	BMSCs	1.3×10^{7} cells	33 days	Better in histological grading but not significant in clinical improvement	[5]
	BMSCs	4×10^{7} cells	-	Improved	[6]
**Osteogenesis imperfecta (OI)**	FMSCs	6×10^{6} cells	-	Improved	[7]
	BMSCs	$5.5\text{-}6.2 \times 10^{8}$ cells	-	Improved	[8]
**Bone Defect (Fracture)**	BMSCs	$2.8 \times 10^{6} – 1.6 \times 10^{7}$ cells	-	Improved	[9]
	BMSCs	2×10^{7} cells	-	Improved	[10]
	BMSCs	1×10^{8} cells	-	Improved and restored limb function	[11]

* Abbreviations: BMSCs – Bone marrow-derived MSCs, ADSCs – Adipose-derived MSCs, FMSCs– Fetal-derived MSCs.

References for supplementary Table 1

[1] Wakitani S, Mitsuoka T, Nakamura N, Toritsuka Y, Nakamura Y, Horibe S (2004). Autologous bone marrow stromal cell transplantation for repair of full-thickness articular cartilage defects in human patellae: two case reports. Cell Transplant, 13:595-600.

[2] Jo CH, Lee YG, Shin WH, Kim H, Chai JW, Jeong EC, *et al.* (2014). Intra-articular injection of mesenchymal stem cells for the treatment of osteoarthritis of the knee: a proof-of-concept clinical trial. Stem Cells, 32:1254-1266.

[3] Kim YS, Choi YJ, Suh DS, Heo DB, Kim YI, Ryu JS, *et al.* (2015). Mesenchymal stem cell implantation in osteoarthritic knees: is fibrin glue effective as a scaffold? Am J Sports Med, 43:176-185.

[4] Davatchi F, Abdollahi BS, Mohyeddin M, Shahram F, Nikbin B (2011). Mesenchymal stem cell therapy for knee osteoarthritis. Preliminary report of four patients. Int J Rheum Dis, 14:211-215.

[5] Wakitani S, Imoto K, Yamamoto T, Saito M, Murata N, Yoneda M (2002). Human autologous culture expanded bone marrow mesenchymal cell transplantation for repair of cartilage defects in osteoarthritic knees. Osteoarthritis Cartilage, 10:199-206.

[6] Vega A, Martin-Ferrero MA, Del Canto F, Alberca M, Garcia V, Munar A, *et al.* (2015). Treatment of Knee Osteoarthritis With Allogeneic Bone Marrow Mesenchymal Stem Cells: A Randomized Controlled Trial. Transplantation, 99:1681-1690.

© 2018. Choi Y et al. Published online at http://www.aginganddisease.org/EN/10.14336/AD.2018.0802

SUPPLEMENTARY DATA

[7] Le Blanc K, Gotherstrom C, Ringden O, Hassan M, McMahon R, Horwitz E, *et al.* (2005). Fetal mesenchymal stem-cell engraftment in bone after in utero transplantation in a patient with severe osteogenesis imperfecta. Transplantation, 79:1607-1614.

[8] Horwitz EM, Prockop DJ, Gordon PL, Koo WW, Fitzpatrick LA, Neel MD, *et al.* (2001). Clinical responses to bone marrow transplantation in children with severe osteogenesis imperfecta. Blood, 97:1227-1231.

[9] Faundez AA, Taylor S, Kaelin AJ (2006). Instrumented fusion of thoracolumbar fracture with type I mineralized collagen matrix combined with autogenous bone marrow as a bone graft substitute: a four-case report. Eur Spine J, 15 Suppl 5:630-635.

[10] Marcacci M, Kon E, Moukhachev V, Lavroukov A, Kutepov S, Quarto R, *et al.* (2007). Stem cells associated with macroporous bioceramics for long bone repair: 6- to 7-year outcome of a pilot clinical study. Tissue Eng, 13:947-955.

[11] Ng MH, Duski S, Tan KK, Yusof MR, Low KC, Rose IM, *et al.* (2014). Repair of segmental load-bearing bone defect by autologous mesenchymal stem cells and plasma-derived fibrin impregnated ceramic block results in early recovery of limb function. Biomed Res Int, 2014:345910.

Supplementary Table 2. The result of the mean and SD of MSC for several passaging.

Time (week)	Passage number	Mean number of counted cells (× 10^4) ± SD	
		Vehicle	RSV
1	1st ~ 2nd passage	1.3 ± 0.40	2.0 ± 0.20
2	3rd ~ 4th passage	18.2 ± 7.20	30.1 ± 11.2
3	5th ~ 6th passage	96.7 ± 32.5	243.1 ± 86.6
4	7th ~ 8th passage	207.6 ± 96.8	1130.6 ± 209
5	9th ~ 10th passage	678.7 ± 96.2	2557.1 ± 613
6	11th ~ 13th passage	1304.1 ± 288	5818.4 ± 921

© 2018. Choi Y et al. Published online at http://www.aginganddisease.org/EN/10.14336/AD.2018.0802
